# Testing the effects of group-affirmation in active conflict: Ukrainians’ trust toward Russia

**DOI:** 10.1371/journal.pone.0270266

**Published:** 2023-05-18

**Authors:** Eunbin Chung, Anna O. Pechenkina

**Affiliations:** 1 Department of Political Science, University of Utah, Salt Lake City, Utah, United States of America; 2 Department of Political Science, Utah State University, Logan, Utah, United States of America; University of Connecticut, UNITED STATES

## Abstract

How can states with a history of recent armed conflict trust one another? Political psychology offers two competing approaches to increase trust between the publics of different countries: appealing to an overarching, common identity above the national level vs. affirming a sense of national identity. This study aims to examine the scope conditions of group-affirmation effects on trust in active conflicts by testing which group-affirmation approach increases trust towards Russia among the Ukrainian public. Distrust between Ukraine and Russia aggravates security fears and limits hope for a meaningful resolution of the bloodiest armed conflict in Europe since 1994. Hostility levels have risen dramatically between the populations of Ukraine and Russia after the events of 2013–2015. The study employs a survey experiment (between-subjects design) to evaluate these competing approaches. The survey was fielded in late May-June 2020 by a reputable public opinion research firm, the Kyiv International Institute of Sociology (KIIS), based in Ukraine. The results suggest that in areas where conflict is salient, national identity affirmation can increase trust in subsamples that hold preexisting baseline levels of affinity toward the outgroup. When combined with the more anti-Russian Ukrainians however, this positive effect was cancelled out. In contrast, emphasis on an overarching, common ingroup identity did not raise trust in any subgroups. Examining the disparate effects of national identity affirmation in anti-Russian and pro-Russian regional subsamples helps specify the scope conditions of which group-affirmation can be most effective.

## Introduction

The impact of group-affirmation has received much attention by social scientists. Namely, psychologists and political scientists have studied the effect of affirming group identities on increasing prosocial attitudes [1, 2], recognition of group-based guilt and shame [3], and reducing prejudice [4] between conflict protagonists in those settings in which the violence has subsided, while some tension or hostility among parties persist. Far less attention has been given to the effectiveness of group-affirmation in areas of high salience and recency of active violence. We conduct a conceptual replication of extant studies on group-affirmation and trust, using similar experimental techniques but applying them to an area of active international conflict. The current debate in the literature juxtaposes two approaches to group identity affirmation. Some scholars argue that affirming one’s national identity that excludes the international rival is more effective in encouraging international trust, while others proffer that affirming an overarching, shared identity that includes the rival country should boost trust more. Both approaches build on the finding that group-level identities may be affirmed just like individual-level identities [5].

We include both approaches as separate treatments in our experiment fielded in Ukraine during May-June 2020. The Ukraine–Russia context represents a difficult case for testing whether group identity affirmation may boost trust. Mutual animosity between Ukrainians and Russians skyrocketed as a result of recent hostilities: the mass protests in Kyiv in late 2013 to early 2014, demanding to replace a pro-Russian government with a staunchly anti-Russian one; Russia then annexed Crimea in March 2014 and fueled the Donbas War in eastern Ukraine in 2014–2015, the bloodiest armed conflict in Europe since the Bosnian War [6]. In early 2020, mutually positive opinions of Russians and Ukrainians dropped by 32–34 percentage points, while mutually negative opinions rose by 28–30 points, compared to September of 2012 (before these events) [7]. Some observers have dubbed this new hostility “the divorce” between Russia and Ukraine [8]. Given democratic politics in Ukraine, its leaders may become hostages of public opinion which views any pro-peace initiatives as appeasement of a hostile neighbor. Can social science offer any solutions to overcome this situation of distrust which inhibits conflict resolution?

The data collected in field experiments conducted over the phone in Ukraine indicate that in the full sample neither identity affirmation treatment impacts trust toward Russia. Yet, affirming Ukrainians’ national identity increased Ukrainians’ trust toward Russia in relatively pro-Russian regions of Ukraine (Southern and Eastern macroregions), while it did not have the same effect in areas where anti-Russian sentiment prevails (Western and Central macroregions). Affirming a common, Eastern Slavic identity did not meaningfully increase Ukrainians’ trust of Russia in any region (the effect is positive but is substantively and statistically negligible). These results help specify the scope conditions of where group-affirmation may be most effective in boosting trust toward an international adversary. They suggest that affirming national identity may be more effective than affirming a common ingroup identity, given that a certain baseline of affinity is present. By implication, using national identity affirmation as a strategy for increasing trust may be more challenging in areas of active violence. Unfortunately, with the 2022 Russian invasion of Ukraine and the extreme antagonism toward Russia that has since unified Ukrainian regions, replicating our results in the Southeast (or previously considered relatively pro-Russian regions) will be challenging.

## Literature review and argument

Several researchers in the social sciences prescribe that a sense of belonging to an overarching, common identity shared across countries generates a sense of commonness that supersedes national identities, helping reconciliation, as it downplays national differences (e.g., [9–11]. For example, the Common Ingroup Identity Model in social psychology claims that recategorization of people from different social groups into a common, superordinate group (that includes both memberships) helps reduce intergroup bias, as former outgroup members are now perceived as ingroup members [10, 12]. In the context of Ukraine and Russia, this approach would emphasize the common East Slavic identity. In contrast to West and South Slavs, East Slavs (the pan-identity for Russians, Ukrainians, and Belarusians) are marked by both their association with the Eastern Orthodox (as opposed to Catholic) Church and the usage of the Cyrillic (as opposed to Latin) alphabet (“Slav” [13]).

This overarching identity stems from the shared history of Kyivan Rus (882–1240), the first East Slavic state that reached its peak in the 11^th^ century (“Kievan Rus” [14]). After the collapse of the Soviet Union, Ukrainian and Russian historiographers tended to disagree about whether the separation of eastern Slavs into three nations began before or after the Mongol invasion of 1240 ([15], p. 125). Despite politicized attempts to usurp the legacy of Kyivan Rus, all three nations trace their origins to the Kyivan project, whose contribution was “the construction of a single identity [that] had a profound impact on the subsequent identities of all the ethnic groups that constituted the Kyivan state, […which] still forms the basis of the cultural commonalities between the three East Slavic nations” ([16], p. 2). Finally, the formation of the East Slav identity preceded the rise of the Tsardom of Russia and later the Russian empire, within which Ukrainians and Belarusians were subordinate to the Russian center. Since the East Slav identity stems from the period of pre-hierarchical relationship with Russia, it lacks the negative connotation in Ukraine.

The conventional approach therefore suggests that placing an emphasis on the easily recognizable East Slavic pan-identity can help overcome the negative effects of strong nationalisms in each country. *This approach predicts that affirming a common (Eastern Slavic) identity tends to increase trust*.

Hypothesis 1: Individuals, whose *overarching*, *shared identity* was affirmed, exhibit more trust toward another country than non-affirmed individuals and individuals with an affirmed national identity.

In contrast, a challenging view argues that affirmation of national identities increases trust between people from different countries [1, 17]. This new approach draws on a set of findings from the psychology of group attachment: in-group affinity does not require hostility toward other groups [18, 19]. That is, the affirmation of national identity is conceptually distinct from chauvinistic appeals to national superiority. This idea is consistent with the theoretical foundation of self-affirmation theory [20] that those who have a clear, secure, and content sense of self tend to be more open, evenhanded, and less defensive toward others. Affirming one’s own social identity has thus been found to increase trust between groups across partisan [21] and racial lines [22], from the perspective of both minority groups [23] and the dominant class [24]. In international relations, if each national population reflects upon the values of their national identity, trust can increase between countries. In the context of Ukraine, this approach would emphasize the national identity of Ukrainians without any allusions or comparisons to Russia. *This approach predicts that affirming a national (Ukrainian) identity tends to increase trust*.

Hypothesis 2: Individuals, whose *national identity* was affirmed, exhibit more trust toward another country than non-affirmed individuals and individuals with an affirmed overarching, shared identity.

## Materials and methods (research design)

### Survey instrument

#### Experimental conditions

The University of Utah Institutional Review Board approved this study (IRB_00131638). Consent was obtained via telephone when KIIS fielded the survey.

To test our hypotheses, we designed a survey experiment with two treatments and a control group, such that 1/3 of the sample received the group-affirmation treatment of the overarching Eastern Slavic identity; another 1/3 received the group-affirmation treatment of the national Ukrainian identity; and the remaining 1/3 served as a control group, which received a question that followed the same structure as the treatment but did not allude to identity, instead asking about dessert preferences (between-subjects design).

The two treatments serve as independent variables of the study.

Treatment 1: Eastern Slavic identity affirmation.

1. There are many positive aspects about being Eastern Slavic. Please choose only one of the following items that you think is the most important value for Eastern Slavs:
  - family; appearance/fashion; patience; working hard; political liberty/democracy
2. Why did you choose the value you chose above as the most important to Eastern Slavs? Why do you think that value is important to Eastern Slavs? Please explain your choice in 1–2 sentences.
  - Open answer
3. How is the value you chose above expressed among Eastern Slavs? Please answer in 1–2 sentences or give an example
  - Open answer
4. Manipulation check: The task on values made me think about:
  - Things Eastern Slavs value about themselves / Things Eastern Slavs do NOT value about themselves.

This manipulation check is a simple yet straightforward way of verifying whether participants were paying attention to the task and thus thinking about values associated with said identity. In addition, this measure has been used as a reliable manipulation check in previous research that uses experimental treatments of identity affirmation [1].

Treatment 2: Ukrainian national identity affirmation.

This treatment is identical to treatment 1, except all instances of “Eastern Slavic or Eastern Slavs” are substituted with “Ukrainian.”

Control.

1. Marmeladki (jellybeans) are a chewy candy. The following is a list of flavors of marmeladki. Please choose only one of the following flavors that you think will be tastiest.
  - Sizzling Cinnamon / Tropical Mango / Apple Jack / Blueberry Balloon / Tutti-Fruitti
2. Please explain why you think the marmeladki (jellybeans) you chose will be tastiest in 1–2 sentences.
  - Open answer
3. When you imagine the taste of the marmeladki (jellybeans) you chose, what do you think it would taste like compared to the others you did not choose? Please explain your choice in 1–2 sentences.
  - Open answer
4. Manipulation check: The task on jellybeans made me think about:
  - Flavors I would like / Flavors I would NOT like

The control task on jellybeans is borrowed from earlier psychological experiments that test the impacts of identity affirmation [25]; this control follows the structure of the treatment, however, is substantively unrelated to values associated with group identities. This control task compares favorably to alternatives. For instance, some studies’ control conditions ask respondents to think about values that are unimportant to their identities. Such a control is unlikely to serve its intended function, since considering values that respondents deem unimportant directly invokes their evaluation of values relevant to the specified identity. In summary, the control task on dessert/candy preferences is substantively irrelevant to treatment conditions yet mimics the structure of the treatment exercise.

Since respondents were randomly assigned to each of the three groups, these groups should be comparable on average on both observable and unobservable characteristics. Thus, any intergroup differences in trust towards Russian government/people should be attributable to the treatment. The control measures (described below) were used to ensure that randomization indeed delivered on average comparable groups of respondents; these results are in the S1 Appendix.

#### Outcomes

Four questions record the dependent variables (all responses are recorded on a 5-item Likert scale): i) how much respondents trust the Russian government; ii) whether respondents believe that the Russian government would exploit Ukraine for its own benefit or treat Ukraine fairly; iii) how much respondents trust the Russian people; iv) how selfish or kind the respondents believe that the Russian people are. These trust questions were adapted from the trust measures in the World Values Survey.

Based on these questions, we construct *Trust toward the Russian government* (a combination of items i and ii, Cronbach’s alpha equals 0.71–0.74; the specified range of Cronbach’s alpha values depends on which responses considered to reflect insufficient attention or effort of respondents are excluded, as we describe below), *Trust toward the Russian people* (a combination of items iii and iv, Cronbach’s alpha equals 0.55–0.68), and overall *Trust toward Russia* (a combination of items i through iv, Cronbach’s alpha equals 0.69–0.71).

Per the preregistered design, we consider alpha above 0.6 as sufficiently high (given a low number of combined items). Additionally, we used principal component analysis to verify that the four dependent measures—designed to capture a single underlying concept of trust—all load on a single factor (shown in the S1 Appendix), since Cronbach’s alpha assumes unidimensionality ([26], p. 102–103).

In addition, the S1 Appendix replicates all analyses using the original four dependent measures and also reports the results for the dependent measures that include “don’t know” responses modeled as a separate answer category.

#### Controls

The survey collected socio-demographic information about respondents as well as their political preferences. The S1 Appendix presents descriptive statistics for all control measures (section 1) and uses these control measures to ensure that randomization was properly done and also includes the descriptive statistics for each control (section 4).

The controls include *Female*, *Age* (a 6-category ordinal variable), *Education* (an 8-category ordinal measure that ranges from elementary to college level or higher), *Russian Ethnicity*, *Settlement Type* (a 7-category ordinal variable whose lowest category indicates a village and the highest category records a city of 500 thousand residents or more), *Professional* (whether a respondent is a white collar professional), *Retiree*, *Income* (a 5-category ordinal variable that ranges from “not enough money to cover food” to “we can afford anything we like”), and *Russian Language* (which indicates that the respondent preferred to answer in Russian). Political preferences are captured by *Russia Thermometer* (a continuous scale that ranges from -50 which indicates a very negative view of Russia to +50 which indicates very positive view of Russia), *Would Not Vote/Would Vote None* (indicates individuals who would abstain from voting or would cross out all parties or refuse to answer if the parliamentary election took place today). We also used *Would Vote for Pro-Zelensky Party* (indicates individuals who would support President Zelensky’s party Servant of the People if the parliamentary election took place today); none of the results change.

### Pre-registered sample size and power analysis

The sample of 2,000 was randomly divided into 3 groups of 675, 663, and 662 individuals each. In phone surveys, the KIIS may only conduct randomization during the survey, so the exact size of each group could not be predetermined.

The power analysis was conducted in the pre-registered report using Soper’s [27, 28] software. For the desired statistical power level of 0.8, the probability level of 0.05, and for 10 predictors (treatment, baseline attitudes towards Russia, ideology, plus a battery of demographic indicators), the required sample size per group should be between 333 and 549 respondents to discern the anticipated effect size of between 0.0305 and 0.0503 respectively (the effect size range is based on previous study by Chung and Woo [17]).

To discern a very small effect size in t-tests (Cohen’s *d* = 0.1), we would need 1,238 respondents per treatment to discern such an effect, i.e., it would be impossible given our group sizes. However, if Cohen’s *d* = 0.2, then our sample size would be sufficient, as only 310 respondents per treatment would be required to uncover such an effect.

### Data inclusion criteria, and regional subsamples

No pilot data were collected for this study. The survey was fielded by KIIS in Ukraine in late May-June 2020 as a phone interview with a random sample of 2,000 individuals. The survey was described as a study on public opinion and included participants in the study if they were over 18 years of age. The KIIS obtained a verbal confirmation of informed consent from respondents. All translation procedures were conducted by the KIIS and verified by the authors.

Using software, the KIIS generated random mobile telephone numbers. After removing non-existing phone numbers, 2,000 phone numbers were randomly selected and contacted. The rate of mobile phone ownership in Ukraine is 96% among adults; furthermore, only 7% of respondents reported that they regularly use a landline phone, and only 1% of respondents reported no access to a mobile phone. These statistics are based on survey results obtained by the KIIS team face-to-face in February 2020.

During data collection, each respondent was randomly assigned into one of three groups, which resulted in 675 respondents assigned to the Eastern Slavic identity treatment, 663 to the national identity treatment, and 662 to the control (in phone surveys, the KIIS conducts randomization during the survey, so the exact size of each group differs slightly).

The KIIS randomized the language in which the enumerators initiated the interview: half of the respondents were greeted in Ukrainian and the other half—in Russian. After hearing the participants’ initial response, the enumerators switched to the language the respondents used. 57.1% of respondents continued the interview in Ukrainian and 42.9% continued the interview in Russian.

Тhe KIIS reweighed the sample based on four attributes (macroregions, type of settlement, age, and gender) in accordance with 2019 data collected by the Central Election Commission of Ukraine and the State Statistics Service of Ukraine. These weights were used in the regression analyses.

There are four well-defined macroregions in Ukraine: West (Chernivetska, Ivano-Frankivska, Khmelnytska, Lvivska, Rivnenska, Ternopilska, Volynska, and Zakarpatska oblasts), Center (Kyiv City as well as Kyyivska, Vinnytska, Zhytomyrska, Kirovohradska, Poltavska, Sumska, Cherkasska, Chernihivska oblasts), South (Dnipropetrovska, Zaporizka, Mykolayivska, Odeska, and Khersonska oblasts), and East (Kharkivska, Donetska, and Luhanska oblasts) [29]. Based on the country’s history and linguistic composition, most residents in the Western and Central regions of Ukraine tend to espouse an “anti-Soviet” identity ([30], p. 151) and to support Ukraine’s integration into the EU and NATO [29]. By contrast, most residents in the Southern and Eastern regions tend to be “Sovietophiles” ([30], p. 151) and tend to oppose Ukraine’s potential joining of the EU and NATO [29].

Besides using macroregions in weights, we return to regional differences to examine subsample-level effects of affirmation (section “Understanding the results: Regional heterogeneity in Ukraine” of the paper).

### Terminating data collection and data exclusion criteria

The data collection was terminated after 2,000 complete survey units were acquired. At the data collection stage, the KIIS only considered whether respondents completed all survey items.

‘Careless or insufficient effort (C/IE)’ responses in surveys are often screened out based on extreme response times and ‘long-string analysis,’ which examines the longest string of identical responses [31–33]. While the former criterion is not applicable in a live phone survey, we screen out the longest strings of identical responses, using the baseline rule of thumb of a conservative cut score as half the scale ([31], p.16). Since most scales used in our survey have 5 response options, individuals who deliver a string of the same response to three or four consecutive Likert items should be removed. The consecutive Likert items appear in the dependent variables of our survey: four outcome questions that measure trust toward Russia all have the same answer options. 230 respondents gave identical answers to three consecutive outcome questions, with 162 of those respondents answering identically to all four questions.

Below we present the most conservative version of analysis where we removed all 230 respondents from the sample as insufficient effort responses. Additional analyses in the S1 Appendix replicate all tests excluding 162 respondents who gave four identical consecutive responses to outcome questions and also not excluding anyone. The results do not change.

## Analysis

The experimental design and procedures were registered on OSF before data collection [34]. A peer-reviewed, registered report protocol of this study was also published in this journal [35]. Replication data are available at Harvard Dataverse: https://doi.org/10.7910/DVN/UX7QMC.

### Positive controls

The survey includes two manipulation checks to measure whether the treatments operated as intended. First, after each treatment, respondents were asked whether the task on values asked subjects to think about things Eastern Slavs (in treatment 1) or Ukrainians (in treatment 2) value about themselves or do not value about themselves. In the control group, the check asked whether the task on jellybeans made them think about flavors they think will be tasty or not tasty. These are tasks that have been used in psychological experiments testing identity affirmation [25].

Below we describe the proportions of those who passed or failed the manipulation checks, while removing careless and insufficient effort (C/IE) responses defined as those who reported three consecutive identical values in the outcome measure. The S1 Appendix also presents the results when removing those who reported four consecutive identical values. Table 1 presents the frequencies of manipulation check passing, failure, and no response using both types of C/IE definitions.

**Table 1:** Breakdown of answers when careless and insufficient effort (C/IE) responses are defined as three consecutive identical values in the outcome measures.

Group	Total assigned	Total quality responses	Passed MC	Failed MC	Hard to say	Refused to answer	No engagement
East Slav identity affirmation	675	598	267	133	120	7	71
Ukrainian identity affirmation	663	582	267	153	100	9	53
Control (jellybeans)	662	590	310	30	66	5	179
Total	2,000	1,770					

Table 1 demonstrates that out of 598 individuals assigned to the overarching identity affirmation treatment (total number of assigned respondents was 675, but 77 subjects were removed as C/IE responses because they provided three identical consecutive values in outcome measures; all other responses are defined as ‘quality responses’), 45% of the quality responses (i.e., those deemed as sufficient effort responses) passed the manipulation check. 22% failed the manipulation check, reporting that the exercise made them think about values that Eastern Slavs do not value about themselves, 20% answered “Hard to say,” 1% said they did not want to answer the question, and the remaining 12% of respondents did not want to engage with the manipulation check at all, keeping quiet.

We observe similar rates among the 582 respondents who received the national identity affirmation treatment (out of 663 subjects assigned to this category, 81 were removed as C/IE responses): 46% of the quality responses passed the manipulation check, 26% failed, 17% answered “Hard to say,” 2% refused to answer, and 9% did not engage with the manipulation check question.

Among the 590 interviewees in the control group (out of 662 subjects assigned to control, 72 were removed as C/IE responses), we observe different rates of response distribution: 53% passed the check, 5% failed, 11% answered “Hard to say,” 1% refused to answer, and 30% did not engage with the manipulation check question.

To summarize, in the two treatment groups, 22–26% of respondents who provided quality responses reported that the treatment made them think about values unimportant to Eastern Slavs and Ukrainians respectively, i.e., they failed the manipulation check. These high failure rates suggest that about a quarter of treated respondents did not react to the treatment exercise in the anticipated way. Additionally, we observe high rates of “hard to say” answers, refusal to answer, and non-engagement with the manipulation check question (combined 28–42% out of quality responses). We revisit this unexpected outcome as well as high rates of various nonresponse in the discussion section by exploring the possible impact of regional heterogeneity in Ukraine.

The second positive control included in the survey assigned half the sample to self-report their attachment to the Ukrainian and Eastern Slavic identity before treatments were administered, while assigning the other half to do so following the treatments. We calculate the difference between ‘before’ and ‘after’ groups within treatment 1, within treatment 2, and within the control group and then compare if the resultant differences are statistically different between treated and control observations by calculating difference-in-differences (DiDs). The results are reported in section 3 of the S1 Appendix. These quantities are not statistically discernible from zero, which means that our confidence that the treatments manipulated one’s attachment to these identities does not increase, however we note that the preregistered design did not calculate power analyses to conduct this positive control check, i.e., we do not have sufficient power to uncover a small effect size when we split the samples into control and treated units). Nonetheless, the DiD analysis indicates that receiving the national identity affirmation (hereafter NIA) treatment moves respondents in the expected direction; the results are not as consistent for the overarching identity affirmation (hereafter OIA).

### Resultant samples and descriptive statistics

Given the heterogeneity of responses to the manipulation check question, there are multiple ways to define which observations are considered “treated.” First, the most restrictive conceptualization of “treated” includes only those respondents who passed the manipulation check. Second, a less restrictive conceptualization of “treated” includes those respondents who passed the manipulation check or answered ‘hard to say.’ Third, the least restrictive definition of ‘treated’ only considers whether a respondent was assigned to a given treatment, regardless of their answer to the manipulation check. Additionally, ‘careless/insufficient effort’ (C/IE) responses may be defined as three or four identical consecutive responses. This yields a total of six samples. The S1 Appendix provides detailed breakdowns for each of these definitions.

The main analysis (included in the paper) reports the results for the subjects who either passed or answered “hard to say” to the MC question (the second definition), and defines C/IE responses as three identical consecutive responses. This sample includes 387, 367, and 376 respondents in the OIA, NIA, and control groups respectively. Table 2 summarizes the descriptive statistics for this sample that we use throughout the main analysis.

**Table 2:** Descriptive statistics.

	count	mean	sd	min	max	sum
	**Dependent variables**					
Trust Russian Gov (2 items)	1089	.1136648	.9562265	-.6674955	3.959325	123.781
Trust Russian Ppl (2 items)	1083	.1879247	.7796197	-1.690631	2.24274	203.5224
Trust (4 items)	1118	.1509776	.7452684	-1.195118	3.959325	168.7929
	**Treatments**					
OIA treatment (def 2)	763	.5072084	.500276	0	1	387
NIA treatment (def 2)	743	.4939435	.5003001	0	1	367
	**Controls**					
Female	1130	1.562832	.4962561	1	2	1766
Age (in years)	1130	46.18673	16.45978	18	92	52191
Education	1120	6.542857	1.575602	1	8	7328
Income	1098	2.606557	.8550524	1	5	2862
Southeast	1130	.3982301	.4897501	0	1	450
Thermometer twd Russia	1051	-.9343482	36.17348	-50	50	-982
Would vote none	1130	.3637168	.4812815	0	1	411
Professional	1130	.2247788	.4176214	0	1	254
Retired	1130	.2415929	.4282383	0	1	273
Settlement Type	1130	4.661062	2.292435	1	7	5267
Russian language	1130	.4504425	.4977583	0	1	509
Russian ethnicity	1130	.0548673	.2278218	0	1	62
Observations	1130					

Based on the pre-registered power analysis (see section ‘Pre-registered sample size and power analysis’), this sample size allows us to uncover a medium effect size in t-tests but not a small effect size.

The S1 Appendix includes all other results. Our results largely do not change based on the definition of “treated.”

Given high rates of failure to pass the manipulation check and high tendency of non-engagement with the manipulation check question, we also include a section—not planned in the preregistered design—that discusses the impact of Ukraine’s regional heterogeneity on these rates.

### Using demographic data to ensure balance on observables

Using sociodemographic data, we compare the means of the treated and control units on observable attributes such as age, gender, education, income, location, and other collected control measures. As the preceding section summarizes, we use three definitions of treatment and two definitions of C/IE responses which produces six samples relative to those in the control group. The main analysis (reported in the paper) uses only one sample of 1,130 respondents (definition 2 of treated and three consecutive identical responses excluded as C/IE). For this sample, all 13 controls are balanced between the treated and control groups. Respondents with more positive views of Russia and older respondents were more likely to pass the MC after the NIA treatment or to say “don’t know.” We make sure to include the imbalanced controls in all regression models.

The S1 Appendix presents the analysis of whether covariates are balanced for all six samples. Section “Randomization” of the S1 Appendix concludes that *Age*, *Education*, *Income*, *Retired*, and *Voted none* are imbalanced under certain definitions.

Additionally, the S1 Appendix also analyzes whether excluded participants differed from included participants in any demographics or primary variables. Sections “Terminating data collection and data exclusion criteria” and “Resultant samples and descriptive statistics” explain the criteria for excluding observations. The results indicate that respondents who were excluded because they gave C/IE answers were quite different from those who gave quality answers: the C/IE observations tend to be men of higher income, those residing in Center-West, less likely to identify Russian as their native language, with a lower baseline affinity toward Russia, and exhibiting lower trust toward Russia. By contrast, those excluded because they failed to pass the manipulation check or refused to engage with treatments did not differ from the rest of the sample in many ways. First, more educated individuals with higher incomes, residing in larger cities were more likely to fail/refuse the MC after the OIA treatment. Second, those residing in the Southeast were more likely to fail/refuse the MC after the NIA treatment. Finally, older, retired individuals with lower incomes exhibiting lower trust toward Russia were more likely to fail/refuse the MC after the control exercise.

### Comparing trust levels by treatment group

The preregistered design states that visualizations of the differences-in-means between treated and control subjects will be done in addition to presenting the results of t-tests. Since the differences-in-means (as described below) are not discernible from 0, we skip the visualization part of the design.

As section “Resultant samples and descriptive statistics” indicates, the strictest conceptualization of treatment does not allow us enough power to obtain differences-in-means. Table 3 presents some of the results of t-test analyses using definition 2 of treatment and defining insufficient effort (C/IE) responses as three identical consecutive values in the outcome measures. In addition, section 5 of the S1 Appendix presents all remaining results of t-tests conducted using definition 2 of treatment–which defines insufficient effort (C/IE) responses as four identical consecutive values in the outcome measures, as well as using all observations assigned to each treatment under both definitions of C/IE responses.

**Table 3:** Differences in trust between treated and control units.

	OIA = 0	OIA = 1	Difference	t-statistic	p-value
Trust Russian Gov (2 items)	0.09	0.13	-0.04	-0.63	0.53
Trust Russian Ppl (2 items)	0.18	0.20	-0.02	-0.34	0.73
Trust (4 items)	0.13	0.17	-0.03	-0.65	0.52
	NIA = 0	NIA = 1	Difference	t-statistic	p-value
Trust Russian Gov (2 items)	0.09	0.12	-0.03	-0.51	0.61
Trust Russian Ppl (2 items)	0.18	0.19	-0.02	-0.29	0.77
Trust (4 items)	0.13	0.16	-0.03	-0.51	0.61

Note: OIA = overarching identity affirmation treatment indicates those respondents whose Eastern Slav identity was affirmed; NIA = national identity affirmation treatment indicates those respondents whose Ukrainian national identity was affirmed. The results shown define treatment based on definition 2, i.e., as those who passed the manipulation check or answered “do not know” after the affirmation/control exercise. Careless or insufficient effort (C/IE) responses are defined as three identical consecutive values in the outcome measures.

Table 3 shows that there are no statistically discernible differences in trust between the subjects treated with the Eastern Slavic identity affirmation and control group (top three rows) as well as between the subjects treated with the Ukrainian national identity affirmation and control group (bottom three rows). Three measures of trust are employed: *Trust toward Russian Government* (index of two survey items), *Trust toward Russian People* (index of two survey items), and *Overall Trust* (index of four survey items). All measures were standardized to have a mean of 0 and a standard deviation of 0.79–0.92. In all tests, the differences are substantively negligible and do not rise to the level of statistical significance. These results are inconsistent with hypotheses 1 and 2. The section on regional heterogeneity of Ukraine explains these results further.

### Individual-level determinants of trust

Table 4 records the impact of overarching identity affirmation (OIA, Models 1–3) and of national identity affirmation (NIA, Models 4–6) on Ukrainians’ trust toward the Russian government (a standardized index of two survey items, Models 1, 4), trust toward Russian people (a standardized index of two survey items, Models 2, 5), and overall trust toward Russia (a standardized index of all four survey items, Models 3, 6). The analyses shown conceptualize treatment as passing the manipulation check or answering “hard to say” after the affirmation/control exercise, and C/IE responses are defined as three consecutive identical values in outcomes. In addition, section 6 of the S1 Appendix presents all remaining regression results conducted using both definitions of treatment as well as using all observations assigned to each treatment under both definitions of C/IE responses.

**Table 4:** The impact of Eastern Slavic identity affirmation and Ukrainian national identity affirmation on trust toward Russian government, Russian people, and overall trust.

	(1)	(2)	(3)	(4)	(5)	(6)
	Trust Gov	Trust Ppl	Trust	Trust Gov	Trust Ppl	Trust
OIA treatment (def 2)	0.0691	0.0277	0.0374			
	(0.084)	(0.064)	(0.057)			
NIA treatment (def 2)				0.0395	0.0124	0.0206
				(0.078)	(0.064)	(0.056)
Female	0.114	0.0497	0.0849	0.136	0.0316	0.0857
	(0.090)	(0.067)	(0.062)	(0.085)	(0.069)	(0.061)
Age (in years)	-0.00179	-0.00303	-0.00176	-0.00135	0.00282	0.00130
	(0.003)	(0.003)	(0.002)	(0.003)	(0.003)	(0.002)
Education	-0.0133	0.0432*	0.0101	-0.00562	0.0313	0.0171
	(0.029)	(0.020)	(0.020)	(0.031)	(0.025)	(0.023)
Income	-0.152 **	-0.0404	-0.0885*	-0.0768	0.0147	-0.0308
	(0.056)	(0.043)	(0.039)	(0.050)	(0.043)	(0.038)
Southeast	0.131	0.138*	0.150*	0.0764	0.141*	0.124
	(0.092)	(0.066)	(0.067)	(0.097)	(0.069)	(0.067)
Thermometer twd Russia	0.00909**	0.00728**	0.00816**	0.00823**	0.00685**	0.00736**
	(0.001)	(0.001)	(0.001)	(0.001)	(0.001)	(0.001)
Would vote none	-0.259**	0.0700	-0.0944	-0.165*	0.0500	-0.0407
	(0.080)	(0.063)	(0.056)	(0.082)	(0.070)	(0.061)
Professional	0.0311	0.0905	0.0516	-0.0451	0.0422	-0.0137
	(0.108)	(0.087)	(0.075)	(0.084)	(0.085)	(0.068)
Retired	0.118	0.160	0.130	0.141	0.118	0.108
	(0.145)	(0.108)	(0.097)	(0.139)	(0.105)	(0.097)
Settlement type	-0.0217	-0.0172	-0.0218	-0.0165	-0.0200	-0.0164
	(0.019)	(0.014)	(0.013)	(0.018)	(0.014)	(0.012)
Russian language	0.279*	0.103	0.178*	0.354**	0.136	0.224**
	(0.112)	(0.070)	(0.075)	(0.122)	(0.073)	(0.079)
Russian ethnicity	0.520**	0.332*	0.371**	0.626**	0.624**	0.625**
	(0.185)	(0.144)	(0.132)	(0.182)	(0.129)	(0.122)
Observations	679	681	693	656	654	669
R2	0.253	0.189	0.294	0.257	0.217	0.306
AIC	1743.0	1466.2	1333.5	1612.1	1409.0	1260.2

Note: OLS regressions coefficients are shown in cells and standard errors—in parentheses.

*p<0.05

**p<0.01. OIA treatment = overarching identity affirmation treatment indicates those respondents whose Eastern Slav identity was affirmed; NIA = national identity affirmation treatment indicates those respondents whose Ukrainian national identity was affirmed. The results shown define treatment as those who passed the manipulation check or answered “do not know” after the affirmation/control exercise. Careless or insufficient effort (C/IE) responses are defined as three identical consecutive values in the outcome measures.

Table 4‘s results may be summarized as: both affirmation treatments have a substantively negligible effect on trust that is not statistically discernible from zero. These results are inconsistent with hypotheses 1 and 2. Therefore, consistent with our analyses of group differences-in-means, we further corroborate the conclusion that neither identity affirmation approach impacted trust when we consider the full sample. We observe the same result in all other samples, as shown in the S1 Appendix.

While the experimental treatments have no impact, baseline attitudes toward Russia measured on a thermometer scale as well as one’s identification as ethnic Russian have strong positive correlations with how much a respondent trusts Russia (the impact of these two measures is statistically discernible in Models 1–6). Furthermore, one’s usage of Russian language during the interview is also positively associated with trust; this effect is statistically discernible in Models 1, 4–6. Residing in the Southeast region of Ukraine is also positively associated with trusting Russia; this effect is statistically meaningful in Models 2, 3, and 5. These results suggest that one’s ethnolinguistic and regional identity as well as preexisting level of affinity toward Russia explain why some respondents trusted Russia more.

We note that the preregistered design specified the usage of ordered logistic regressions, but since we combined items into continuous standardized measures of trust, OLS is a more appropriate tool. The S1 Appendix includes ordered logistic regressions using raw 5-category outcome survey items as robustness analyses; all results are consistent with those reported here, i.e., in the full sample neither identity affirmation treatment impacts trust toward Russia.

To summarize, we have followed the preregistered design to uncover no differences in trust between individuals whose Eastern Slavic identity or Ukrainian national identity was affirmed and those who received the control exercise; we conclude that in the full sample, the affirmation treatments produced no impact on the extent to which Ukrainians trust Russia, which is inconsistent with our expectations. The next section was unplanned in the preregistered design; however, we include it as exploratory analysis to probe why we see no differences in the full sample.

### Understanding the results: Regional heterogeneity in Ukraine

#### Regional disparity in the impacts of group identity affirmations

This section presents exploratory analyses of the effects of Eastern Slavic identity affirmation and of Ukrainian national identity affirmation on trust in regional subsamples of Ukrainians; this section was not preregistered, and we offer it to suggest a way to understand our results.

As described in the section on control measures, Ukraine has distinct macroregions: West, Center, South, and East. Different trajectories of historical development between Center-West on the one hand versus Southeast on the other hand have produced different ethno-linguistic composition (Southeast has more Russian speakers) as well as different political preferences (more than half of Center-West respondents supported the EU and NATO integration in 2015, while less than half did so in the Southeast) [29].

Due to these patterns, we obtain differences-in-means in trust between treated and control subjects in the two subsamples of West-Center vs. Southeast. These results are summarized in Table 5. Section 8 of the S1 Appendix also presents the results for samples in which careless or insufficient effort (C/IE) responses are defined as four identical consecutive values in the outcome measures and defining treatment as all assigned subjects.

**Table 5:** Regional differences in trust between treated and control units.

	OIA = 0	OIA = 1	Difference	t-statistic	p-value
Trust Russian Gov (West-Center)	-0.03	-0.02	-0.01	-0.10	0.92
Trust Russian Gov (Southeast)	0.26	0.40	-0.13	-1.03	0.30
Trust Russian Ppl (West-Center)	0.06	0.07	-0.01	-0.20	0.84
Trust Russian Ppl (Southeast)	0.35	0.39	-0.04	-0.41	0.68
Trust (4 items, West-Center)	0.02	0.01	0.00	0.03	0.98
Trust (4 items, Southeast)	0.30	0.40	-0.10	-1.06	0.29
	NIA = 0	NIA = 1	Difference	t-statistic	p-value
Trust Russian Gov (West-Center)	-0.03	-0.16	0.12	1.72	0.09
Trust Russian Gov (Southeast)	0.26	0.56	-0.30	-2.40	0.02
Trust Russian Ppl (West-Center)	0.06	0.01	0.05	0.70	0.48
Trust Russian Ppl (Southeast)	0.35	0.48	-0.13	-1.30	0.19
Trust (4 items, West-Center)	0.02	-0.08	0.09	1.68	0.09
Trust (4 items, Southeast)	0.30	0.52	-0.22	-2.30	0.02

Note: OIA = overarching identity affirmation treatment indicates those respondents whose Eastern Slav identity was affirmed; NIA = national identity affirmation treatment indicates those respondents whose Ukrainian national identity was affirmed. The results shown define treatment based on definition 2, i.e., as those who passed the manipulation check or answered “do not know’” after the affirmation/control exercise. Careless or insufficient effort (C/IE) responses are defined as three identical consecutive values in the outcome measures.

The impact of the overarching identity affirmation (OIA) produces no effect on trust toward Russia in both Center-West and Southeast regions.

Table 5 also reports the impacts of NIA on trust measures in regional subsamples. In contrast to the expectation, the NIA treatment decreases trust by 0.09–0.12 units (the effects are significant in one-tail test, p = 0.09) toward government and overall trust in the Center-West region and increases trust toward government and overall trust by 0.22–0.30 units (p = 0.02) in the Southeast. The NIA treatment makes no difference on trust toward people.

Table 5 reveals that the control group’s baseline levels of trust toward Russia in West-Center are 0.28 units lower than control group’s trust in the Southeast. Furthermore, if we compare the reported levels of trust among treated subjects between regions, we observe that trust toward Russia is higher among treated subjects in the Southeast by 0.44 to 0.72 units. This means that exposure to the Ukrainian national identity affirmation generates the expected effect of increasing trust toward Russian government and overall trust among individuals who have a priori pro-Russian baseline views. Among individuals who have anti-Russian baseline views, the NIA treatment decreases trust toward government and overall trust (in a one-tail test). To sum up, the negligible substantive effects of the NIA treatment on trust observed in the full sample (Table 6) appear to be the result of disparate effects that NIA has in West-Center vs. Southeast.

**Table 6:** Regional differences in failure/refusal to engage with treatments.

	West-Center	Southeast	Difference	t	p-value
Failure/Refusal to engage w/OIA	0.38	0.31	0.06	1.54	0.12
Failure/Refusal to engage w/NIA	0.40	0.51	-0.11	-2.49	0.01
Failure/Refusal to engage w/Control	0.37	0.36	0.01	0.28	0.78

Note: OIA = overarching Eastern Slavic identity affirmation treatment; NIA = national Ukrainian identity affirmation treatment. Failure/refusal to engage with treatments/control is coded as 1 if the respondent answered that the affirmation exercise made them think about values unimportant to Eastern Slavs/Ukrainians or if the respondent explicitly said ‘refuse to answer’ or if the respondent kept quiet during the manipulation check question. Failure/refusal to engage with treatments/control is coded as 0 if the respondent passed the manipulation check or answered, ‘hard to say.’ Careless or insufficient effort (C/IE) responses are defined as three identical consecutive values in the outcome measures.

#### High failure rates

We observe high failure rates and nonengagement with the manipulation check question in our experiment. To understand if there were any patterns in the data regarding failure/refusal to engage with treatments, we created binary indicators of failure/refusal to engage with treatments/control. These indicators are coded as 1 if the respondent answered that the affirmation exercise made them think about values unimportant to Eastern Slavs/Ukrainians (or jellybean flavors that would not be tasty in the control group) or if the respondent explicitly said ‘refuse to answer’ or kept quiet during the manipulation check question. To keep the analyses presented in the paper consistent, the indicators of failure/refusal to engage with treatments/control are coded as 0 if the respondent passed the manipulation check or said ‘hard to say.’ Section 8.2.3 of the S1 Appendix also presents the results for the indicators that are coded 0 only if the respondent passed the manipulation check. All raw frequencies of answers to the manipulation check questions are shown in Table 1.

We examine trust measures and Russia thermometer between individuals who did and did not fail the manipulation check (or refused treatments or control). There are no differences between those groups in the full sample as well as in the regional subsamples.

Table 6 presents differences in the proportions of respondents who failed the manipulation check or refused to engage with treatments or control by region. First, we observe that there is no region-specific pattern in failure/refusal to engage with the OIA treatment or the control exercise on jellybeans.

By contrast, region-specific patterns emerge when we consider failure/refusal in the NIA treatment groups: The refusal/failure rate to engage with the NIA exercise rate is 11 percentage points higher in the Southeast than in the Center-West. This pattern suggests that the NIA exercise made more respondents uncomfortable in the Southeast, however given no differences in trust/affinity toward Russia levels, it is unclear what determined higher failure/refusal to engage in the Southeast.

## Discussion

### Summary of findings

This project asks what type of identity affirmation is more likely to increase the extent to which Ukrainians trust Russians. We draw on two competing theoretical approaches to overcoming distrust—a common, supranational identity affirmation vs. national identity affirmation—to answer this question. We have followed the preregistered design [35] to uncover no differences in trust between individuals whose Eastern Slavic identity or Ukrainian national identity was affirmed and those who received the control exercise. We conclude that in the full sample, the affirmation treatments produced no impact on the extent to which Ukrainians trust Russians, which is inconsistent with our expectations.

However, our additional analyses suggest that regional heterogeneity of Ukraine is likely responsible for the null results of the NIA treatment in the full sample. These findings imply that the effects of identity affirmation on attitudes toward adversarial outgroups may depend on prior sentiments. Center-West vs. Southeast macroregions differ substantially in their levels of baseline affinity and trust toward Russia. We find that the national identity affirmation (NIA) treatment boosts *Trust toward Russian Government* and *Overall Trust* in the pro-Russian Southeast. By contrast, the NIA treatment decreases trust toward government and overall trust (one-tail test) in the more anti-Russian Center-West region. These are exploratory results, which is why we encourage future research to identify further scope conditions under which the NIA treatment does and does not increase prosocial attitudes toward adversaries.

#### Contribution

Whether societies can overcome trauma that stems from armed conflict impacts the stability of international interactions among states. This research tested whether emphasizing national identity (as opposed to a larger, common identity shared with rivals) in post-conflict societies can help overcome distrust of former adversaries, a question of both academic and policy interest. Strong national identities and nationalisms often carry bad reputations, even referred to as “ideological poison” in a 2019 speech by German President Frank-Walter Steinmeier [36, 37]. Undoubtedly, vehement nationalist outcries have fostered blind loyalty to one’s country at the expense of outgroups. In contrast to this common view of nationalism, however, we emphasize the need to differentiate an inward-looking sense of benign affection for one’s nation from the more narrow-minded, immoral sense of nationalism that involves superiority over another nation.

In fact, as we argue, there are theoretical reasons to expect that promoting an inward-looking sense of attachment that affirms the positive aspects of one’s national identity could increase trust toward an outgroup. Previous research has found that affirming national identities can increase prosocial attitudes between countries where violence has subsided decades ago, but some tension and underlying animosity remains [1]. Strikingly, we find that national identity affirmation can potentially increase trust toward an adversary even in a context where the memory of violence is fresh. A caveat here is that affirming national identity was more effective when subjects already held a baseline level of affinity toward the adversary.

We also test the conventional view that affirming a common identity increases trust toward outgroup. The Common Ingroup Identity Model in social psychology maintains that encouraging people to recategorize and identify themselves with a broader, superordinate social category that includes outgroup members mitigates bias toward the outgroup [10]. The model holds many parallels with other studies in social sciences, which agree that supranational, homogenous global identities will become more important than national identities with continued globalization [38]. However, as we found, emphasizing a common, East Slavic identity that included Russia did not have an effect of increasing Ukrainians’ trust of Russia.

Our results contribute to the study of identity affirmation and trust in several ways. First, they help specify the scope conditions of group-affirmation and the contexts in which they can be most effectively utilized. The fact that national identity affirmation was found to increase prosociality in Northeast Asia (between Japan, China, and South Korea) in existing research [39], as well as the finding in this study that affirming Ukrainians’ national identity boosted their trust toward the Russian government in relatively pro-Russian regions, similarly suggest that national identity affirmation may be effective where a minimum baseline of security is obtained amidst persisting tension, but can relatively lose its power in areas of extreme hatred and ongoing animosity. These results suggest that in these areas the effects of NIA might backfire. Furthermore, elsewhere, we examine the potential mechanism behind the negative impact that national identity affirmation has on trust in the anti-Russian region and its positive impact in the pro-Russian region of Ukraine in 2020. We find that this result is driven by individuals with above-average levels of attachment to Ukrainian identity or even a sense of Ukrainian superiority. This heightened attachment to Ukrainian identity likely makes the experience of the national identity treatment more conflicting for the individuals’ worldview if they reside in the West-Center and less conflicting if they reside in the Southeast due to negative or positive baseline respective attitudes towards Russia [40].

The hardest case for identity affirmation as a technique to improve intergroup relations would be in a context where conflict is ongoing, and antagonism prevails. Considering the intensity of recurring hostilities of the Donbas War where there are still multiple ceasefire violations every month by the time the survey was fielded in May-June 2020, we have tested the impact of identity affirmations on trust in a difficult test case. In addition, the power disparity between Ukraine and Russia makes it easier for Ukrainians to view Russia in an enemy rather than an ally image (see [41] for image prototypes in image theory). Surprisingly, we find that even in this challenging test case, national identity affirmation did increase Ukrainians’ trust toward Russian government in 40% of the sample; namely among people who already held some baseline level of affinity toward Russia.

#### Additional potential determinants of higher failure/refusal to engage rates

Another difference that stands out in this study when compared to existing research is its methodology. Namely, this study was conducted via a mobile phone interview, whereas previous studies on group-affirmation generally took the form of lab or lab-in-the-field experiments where subjects participated via a computer-based survey. The format of a phone interview may have affected the attention of respondents in various ways, as suggested by the high rate of those failing to pass the manipulation check, and a large proportion of respondents who decided against engaging with certain questions at all. While phone interviews may be efficient in collecting a random sample, phone interviews may be more vulnerable to social desirability bias, due to lower (perceived) anonymity. In addition, respondents in phone surveys have higher nonresponse rates and are more prone to be influenced by the interviewer (such as voice and tone), in terms of both the decision to participate and when giving their answers [42]. Responses in phone interviews have also been found to be relatively more sensitive to factors such as the length of the survey (e.g., [43]) and the topic of the survey (e.g., [44]). From the nature of the device, phone respondents may be in the middle of some other task at the time of contact, leading to higher risk of distraction, low attention, short concentration spans, and lower preparedness compared to computer-based surveys. For this reason, responses may be shorter and faster [45, 46]. If these are made more hastily, there is a chance that responses could also be based on less information and heuristic-based, superficial processing [47–49]. In short, our findings may have been weakened by reasons for nonresponse that were less related to the survey and more to situational factors.

## Supporting information

S1 Appendix Online appendix for testing the effects of group-affirmation in active conflict: Ukrainians’ trust toward Russia. (PDF)
