# Supplementary material for: Testing the effects of group-affirmation in active conflict: Ukrainians’ trust toward Russia
Source: PLoS One. 2023 May 18;18(5):e0270266. doi: 10.1371/journal.pone.0270266 (PMC10194885; doi:10.1371/journal.pone.0270266)
Supplement: S1 Appendix — (PDF) [file pone.0270266.s001.pdf]

Online Appendix for
Testing the effects of group-affirmation in active conflict: Ukrainians' trust toward Russia

Eunbin Chung*†   Anna O. Pechenkina‡

April, 2022

Contents

1 Descriptive Statistics **7**

2 Unidimensionality of outcome measures **9**

3 Positive control: Attachment to Eastern Slavic identity and to Ukrainian national identity measured before and after treatment/control exercises **11**

4 Breakdown of answers when careless and insufficient effort (C/IE) responses are defined as four identical values in the outcome measures **13**

5 Resultant samples and power of the test **14**

6 Randomization: Are covariates balanced between treated and control groups? **16**
6.1 Summary of randomization analyses 16
6.2 Removing 'careless/insufficient effort' (C/IE) responses as *three* consecutive answers of the same value 17
6.3 Removing 'careless/insufficient effort' (C/IE) responses as *four* consecutive answers of the same value 21

7 Analyses: T-tests **25**
7.1 Effects of the OIA treatment on trust 25
7.1.1 Treatment conceptualized as definition 2: Those who passed the MC or said "hard to say" 25
7.1.2 Treatment conceptualized as everyone assigned to OIA 26
7.2 Effects of the NIA treatment on trust 26
7.2.1 The NIA treatment conceptualized as definition 2: Those who passed the MC or said "hard to say" 26

*Names appear alphabetically.
†University of Utah, eunbin.chung@utah.edu
‡Utah State University, anna.pechenkina@usu.edu

7.2.2 Treatment conceptualized as everyone assigned to NIA 28

8 Analysis: Regression 29

8.1 The impact of OIA 29

8.2 The impact of NIA 36

9 Analysis: Why were some respondents more likely to answer 'Hard to say' to trust questions? 43

10 Analysis: Understanding the results through regional heterogeneity in Ukraine 48

10.1 Region-level effects of the OIA treatment on trust 48

10.1.1 The OIA treatment conceptualized based on definition 2: Those who passed the MC or said "hard to say" 48

10.1.2 Treatment conceptualized as everyone assigned to OIA 49

10.2 Region-level effects of the NIA treatment on trust 50

10.2.1 The NIA treatment conceptualized based on definition 2: Those who passed the MC or said "hard to say" 50

10.2.2 Treatment conceptualized as everyone assigned to NIA 51

10.2.3 Regional differences in failure/refusal to engage with treatments 52

11 How the KIIS determines convenient language of communication 53

12 Randomization: Are primary variables and demographics balanced between included and excluded observations? 54

List of Tables

A1 Descriptive statistics: All variables used in analyses (C/IE responses are defined as *three* consecutive identical values in outcomes) 7

A2 Descriptive statistics: All variables used in analyses (C/IE responses are defined as *four* consecutive identical values in outcomes) 8

A3 Principal component analysis of two outcome measures combined into *Trust toward Russian Government* 9

A4 Principal component analysis of two outcome measures combined into *Trust toward Russian People* 9

A5 Principal component analysis of four outcome measures combined into *Overall Trust* 10

A6 Differences in Ukrainian national identity attachment between those who were asked before and after the Ukrainian national identity affirmation/control exercise 11

A7 Differences in Eastern Slavic identity attachment between those who were asked before and after the Eastern Slav identity affirmation/control exercise 12

A8 Breakdown of answers when careless and insufficient effort (C/IE) responses are defined as four identical values in the outcome measures 13

A9 Conceptualizations of treated when careless and insufficient effort (C/IE) responses are defined as three identical consecutive values in the outcome measures 14

A10 Conceptualizations of treated when careless and insufficient effort (C/IE) responses are defined as four identical consecutive values in the outcome measures 14
A11 Summary of randomization analyses 16
A12 Differences in observable attributes between those who passed the manipulation check after the Eastern Slav identity affirmation and those who passed the manipulation check after the control exercise (OIA, definition 1) 17
A13 Differences in observable attributes between those who passed the manipulation check or answered "hard to say" after the Eastern Slav identity affirmation and those who passed the manipulation check or answered "hard to say" after the control exercise (OIA, definition 2) 18
A14 Differences in observable attributes between those who assigned to the Eastern Slav identity affirmation treatment and those who were assigned to the control exercise (OIA, definition 3) 18
A15 Differences in observable attributes between those who passed the manipulation check after the Ukrainian national identity affirmation and those who passed the manipulation check after the control exercise (NIA, definition 1) 19
A16 Differences in observable attributes between those who passed the manipulation check or answered "hard to say" after the Ukrainian national identity affirmation and those who passed the manipulation check or answered "hard to say" after the control exercise (NIA, definition 2) 19
A17 Differences in observable attributes between those who assigned to the Ukrainian national identity affirmation treatment and those who were assigned to the control exercise (NIA, definition 3) 20
A18 Differences in observable attributes between those who passed the manipulation check after the Eastern Slav identity affirmation and those who passed the manipulation check after the control exercise (OIA, definition 1; C/IE = 4 consecutive) 21
A19 Differences in observable attributes between those who passed the manipulation check or answered "hard to say" after the Eastern Slav identity affirmation and those who passed the manipulation check or answered "hard to say" after the control exercise (OIA, definition 2; C/IE = 4 consecutive) 22
A20 Differences in observable attributes between those who assigned to the Eastern Slav identity affirmation treatment and those who were assigned to the control exercise (OIA, definition 3; C/IE = 4 consecutive) 22
A21 Differences in observable attributes between those who passed the manipulation check after the Ukrainian national identity affirmation and those who passed the manipulation check after the control exercise (NIA, definition 1; C/IE = 4 consecutive) 23
A22 Differences in observable attributes between those who passed the manipulation check or answered "hard to say" after the Ukrainian national identity affirmation and those who passed the manipulation check or answered "hard to say" after the control exercise (NIA, definition 2; C/IE = 4 consecutive) 23
A23 Differences in observable attributes between those who assigned to the Ukrainian national identity affirmation treatment and those who were assigned to the control exercise (NIA, definition 3; C/IE = 4 consecutive) 24

A24 Differences in trust between those who passed the MC or said "hard to say" after the Eastern Slav identity affirmation treatment and those who passed the MC or said "hard to say" after the control exercise (OIA, definition 2; C/IE = 3 consecutive) 25
A25 Differences in trust between those who passed the MC or said "hard to say" after the Eastern Slav identity affirmation treatment and those who passed the MC or said "hard to say" after the control exercise (OIA, definition 2; C/IE = 4 consecutive) 25
A26 Differences in trust between those assigned to the Eastern Slav identity affirmation treatment and those assigned to the control exercise (OIA, all assigned; C/IE = 3 consecutive) 26
A27 Differences in trust between those assigned to the Eastern Slav identity affirmation treatment and those assigned to the control exercise (OIA, all assigned; C/IE = 4 consecutive) 26
A28 Differences in trust between those who passed the MC or said "hard to say" after the Ukrainian national identity affirmation treatment and those who passed the MC or said "hard to say" after the control exercise (NIA, definition 2; C/IE = 3 consecutive) 26
A29 Differences in trust between those who passed the MC or said "hard to say" after the Ukrainian national identity affirmation treatment and those who passed the MC or said "hard to say" after the control exercise (NIA, definition 2; C/IE = 4 consecutive) 27
A30 Differences in trust between those assigned to the Ukrainian national identity affirmation treatment and those assigned to the control exercise (NIA, all assigned; C/IE = 3 consecutive) 28
A31 Differences in trust between those assigned to the Ukrainian national identity affirmation treatment and those assigned to the control exercise (NIA, all assigned; C/IE = 4 consecutive) 28
A32 The impact of overarching identity affirmation (OIA) on Ukrainians' trust toward Russia 30
A33 The impact of overarching identity affirmation (OIA) on Ukrainians' trust toward Russia 31
A34 The impact of overarching identity affirmation (OIA) on Ukrainians' trust toward Russia; trust measured as raw items 32
A35 The impact of overarching identity affirmation (OIA) on Ukrainians' trust toward Russia; trust measured as raw items 33
A36 The impact of overarching identity affirmation (OIA) on Ukrainians' trust toward Russia; trust measured as raw items 34
A37 The impact of overarching identity affirmation (OIA) on Ukrainians' trust toward Russia; trust measured as raw items 35
A38 The impact of national identity affirmation (NIA) on Ukrainians' trust toward Russia 37
A39 The impact of national identity affirmation (NIA) on Ukrainians' trust toward Russia 38
A40 The impact of national identity affirmation (NIA) on Ukrainians' trust toward Russia; trust measured as raw items 39
A41 The impact of national identity affirmation (NIA) on Ukrainians' trust toward Russia; trust measured as raw items 40

A42 The impact of national identity affirmation (NIA) on Ukrainians' trust toward Russia; trust measured as raw items 41
A43 The impact of national identity affirmation (NIA) on Ukrainians' trust toward Russia; trust measured as raw items 42
A44 Descriptive statistics for dichotomous dependent variables where answer 'Hard to say' is coded as '1' 43
A45 Descriptive statistics for dichotomous dependent variables where answer 'Hard to say' is coded as '1' 43
A46 The impact of socio-economic covariates on respondents' tendency to answer 'hard to say' when answering trust questions 44
A47 The impact of treatments (definition 1) and socio-economic covariates on respondents' tendency to answer 'hard to say' when answering trust questions 45
A48 The impact of treatments (definition 2) and socio-economic covariates on respondents' tendency to answer 'hard to say' when answering trust questions 46
A49 The impact of being assigned to a certain treatment and socio-economic covariates on respondents' tendency to answer 'hard to say' when answering trust questions 47
A50 Differences in trust between those who passed the MC or said "hard to say" after the Eastern Slav identity affirmation treatment/the control exercise (OIA, definition 2; C/IE = 4 consecutive) 48
A51 Differences in trust between those assigned to the Eastern Slav identity affirmation treatment and those assigned to the control exercise (OIA, all assigned; C/IE = 3 consecutive) 49
A52 Differences in trust between those assigned to the Eastern Slav identity affirmation treatment and those assigned to the control exercise (OIA, all assigned; C/IE = 4 consecutive) 49
A53 Differences in trust between those who passed the MC or said "hard to say" after the Ukrainian national identity affirmation treatment and those who were assigned to the control exercise (NIA, definition 2; C/IE = 4 consecutive) 50
A54 Differences in trust between those assigned to the Ukrainian national identity affirmation treatment and those assigned to the control exercise (NIA, all assigned; C/IE = 3 consecutive) 51
A55 Differences in trust between those assigned to the Ukrainian national identity affirmation treatment and those assigned to the control exercise (NIA, all assigned; C/IE = 4 consecutive) 51
A56 Regional differences in failure/refusal to engage with treatments 52
A57 Differences between quality and careless and insufficient effort (C/IE) observations 54
A58 Differences between quality and careless and insufficient effort (C/IE) observations 55
A59 Differences between respondents who passed/engaged with the MC after the OIA treatment and those who failed the OIA treatment or refused to engage with the MC after the OIA treatment 55
A60 Differences between respondents who passed/engaged with the MC after the NIA treatment and those who failed the NIA treatment or refused to engage with the MC after the NIA treatment 56

A61 Differences between respondents who passed/engaged with the MC after control and those who failed the MC after control or refused to engage with the MC after control . . . 56

1 Descriptive Statistics

Table A1: Descriptive statistics: All variables used in analyses (C/IE responses are defined as *three* consecutive identical values in outcomes)

	count	mean	sd	min	max	sum
**Dependent variables**						
Trust Russian Gov (2 items)	1669	.0208856	.9245357	-.7402256	3.726597	34.85801
Trust Russian Ppl (2 items)	1645	.1833751	.7771946	-1.690631	2.24274	301.6521
Trust	1716	.1449903	.7459287	-1.690631	3.959325	248.8034
**Treatments**						
OIA treatment (def 1)	577	.4627383	.4990423	0	1	267
OIA treatment (def 2)	763	.5072084	.500276	0	1	387
Assigned to OIA treatment	1188	.503367	.5001992	0	1	598
NIA treatment (def 1)	577	.4627383	.4990423	0	1	267
NIA treatment (def 2)	743	.4939435	.5003001	0	1	367
Assigned to NIA treatment	1172	.496587	.5002018	0	1	582
**Controls**						
Female	1770	1.551977	.4974315	1	2	2747
Age (in years)	1770	47.10395	16.39667	18	92	83374
Education	1750	6.588	1.561023	1	8	11529
Income	1706	2.600234	.8723044	1	5	4436
Southeast	1770	.4022599	.4904924	0	1	712
Thermometer twd Russia	1589	-.5066079	36.32696	-50	50	-805
Would vote for pro-Zelensky party	1770	.1728814	.3782515	0	1	306
Would vote none	1770	.3785311	.485158	0	1	670
Professional	1770	.2265537	.4187197	0	1	401
Retired	1770	.2531073	.4349148	0	1	448
Settlement Type	1770	4.800565	2.255901	1	7	8497
Russian language	1770	.4050847	.4910472	0	1	717
Russian ethnicity	1770	.0553672	.2287603	0	1	98
Observations	1770					

Table A2: Descriptive statistics: All variables used in analyses (C/IE responses are defined as *four* consecutive identical values in outcomes)

	count	mean	sd	min	max	sum
**Dependent variables**						
Trust Russian government (2 items)	1669	.0208856	.9245357	-.7402256	3.726597	34.85801
Trust Russian people (2 items)	1713	.1360428	.8039223	-1.690631	2.24274	233.0413
Trust	1784	.1108097	.7528178	-1.690631	3.959325	197.6844
**Treatments**						
OIA treatment (def 1)	599	.4657763	.4992443	0	1	279
OIA treatment (def 2)	789	.5069708	.5002685	0	1	400
Assigned to OIA treatment	1233	.5004055	.5002027	0	1	617
NIA treatment (def 1)	597	.4639866	.4991195	0	1	277
NIA treatment (def 2)	770	.4948052	.500298	0	1	381
Assigned to NIA treatment	1221	.4954955	.5001846	0	1	605
**Controls**						
Female	1838	1.550054	.4976236	1	2	2849
Age (in years)	1838	47.10446	16.42707	18	92	86578
Education	1817	6.571822	1.573262	1	8	11941
Income	1770	2.602825	.8735113	1	5	4607
Southeast	1838	.3966268	.4893304	0	1	729
Thermometer twd Russia	1652	-1.313559	36.36899	-50	50	-2170
Would vote for pro-Zelensky party	1838	.1741023	.3793006	0	1	320
Would vote none	1838	.3775843	.4849148	0	1	694
Professional	1838	.2257889	.4182146	0	1	415
Retired	1838	.2519042	.4342247	0	1	463
Settlement Type	1838	4.796518	2.252428	1	7	8816
Russian language	1838	.3998912	.490009	0	1	735
Russian ethnicity	1838	.0538629	.2258084	0	1	99
Observations	1838					

2 Unidimensionality of outcome measures

Table A3: Principal component analysis of two outcome measures combined into *Trust toward Russian Government*

Factor	Eigenvalue	Difference	Proportion	Cumulative
Factor1	1.60509	1.21018	0.8025	0.8025
Factor2	0.39491	.	0.1975	1.0000
Retained factors	1			

Note: Number of obs = 1,693; LR test: independent vs. saturated: chi2(1) = 771.18 Prob>chi2 = 0.0000. The two questions combined into the index *Trust toward Russian Government* include: "Do you trust Russian government?" and "Do you think Russian government will treat Ukraine fairly?"

Table A4: Principal component analysis of two outcome measures combined into *Trust toward Russian People*

Factor	Eigenvalue	Difference	Proportion	Cumulative
Factor1	1.51167	1.02335	0.7558	0.7558
Factor2	0.48833	.	0.2442	1.0000
Retained factors	1			

Note: Number of obs = 1,651; LR test: independent vs. saturated: chi2(1) = 500.72 Prob>chi2 = 0.0000. The two questions combined into the index *Trust toward Russian People* include: "Do you trust Russian people?" and "Do you think Russian people are selfish or kind?"

Table A5: Principal component analysis of four outcome measures combined into *Overall Trust*

Factor	Eigenvalue	Difference	Proportion	Cumulative
Factor1	2.35117	1.54437	0.5878	0.5878
Factor2	0.80679	0.29857	0.2017	0.7895
Factor3	0.50822	0.17441	0.1271	0.9165
Factor4	0.33382	.	0.0835	1.0000
Retained factors	1			

Note: Number of obs = 1,507; LR test: independent vs. saturated: chi2(1) = 1706.14 Prob>chi2 = 0.0000. The four questions combined into the index *Overall Trust* include: "Do you trust Russian government?", "Do you think Russian government will treat Ukraine fairly or will exploit Ukraine?", "Do you trust Russian people?" and "Do you think Russian people are selfish or kind?"

3 Positive control: Attachment to Eastern Slavic identity and to Ukrainian national identity measured before and after treatment/control exercises

We calculate the difference between 'before' and 'after' groups within treatment 1, within treatment 2, and within the control group and then compare if the resultant differences are statistically different between treated and control observations by calculating differences-in-differences (DiDs). The results are reported in section 3 of the appendix. These quantities are not statistically discernible from zero (however, we do not have sufficient power to uncover a small effect size when we split the samples into control and treated units). Nonetheless, the DiD analysis indicates that receiving the treatment moves respondents who were affirmed of their national identity in the expected direction.

Table A6 demonstrates that although all subjects' national identity attachment declines as a result of taking the survey, i.e., respondents assigned to describe their national attachment *after* the national identity affirmation or control exercise reported lower levels of attachment than those assigned to answer this question *before* the exercise. However, the treated subjects' attachment declines by a smaller amount than that of the control subjects. It is unclear why taking the survey lowered respondents' attachment to national identity, however the fact that exposure to national identity affirmation reduced this effect is consistent with our expectations.

Table A7 shows that we observe the same pattern among those who received the overarching identity affirmation using the strictest definition of treatment, but not when we use the second definition or examine the before and after patterns among treated and control subjects assigned to the OIA group.

In summary, since the obtained differences-in-differences are not statistically discernible, our confidence that the treatments manipulated one's attachment to these identities does not increase, yet since most obtained DiDs indicate movement in the expected direction, our confidence is not undermined by these patterns.

Table A6: Differences in Ukrainian national identity attachment between those who were asked before and after the Ukrainian national identity affirmation/control exercise

	Before	After	Diff	se	t-statistic	p-value	DiD	se
NIA treated (def 1)	0.08	-0.01	0.09	0.09	1.01	0.31	-0.02	0.13
NIA control (def 1)	0.00	-0.11	0.11	0.10	1.14	0.26		
NIA treated (def 2)	0.06	0.00	0.05	0.08	0.68	0.49	-0.06	0.12
NIA control (def 2)	0.04	-0.07	0.11	0.09	1.25	0.21		
Assigned to NIA	0.02	-0.05	0.07	0.07	1.08	0.28	-0.04	0.10
Assigned to control	0.08	-0.01	0.09	0.07	1.26	0.21		

Table A7: Differences in Eastern Slavic identity attachment between those who were asked before and after the Eastern Slav identity affirmation/control exercise

	Before	After	Diff	se	t-statistic	p-value	DiD	se
OIA treated (def 1)	-0.02	0.00	-0.03	0.10	0.79	0.79	-0.02	0.14
OIA control (def 1)	-0.10	-0.09	-0.01	0.11	-0.08	0.93		
OIA treated (def 2)	0.02	0.03	-0.01	0.09	-0.15	0.88	0.04	0.13
OIA control (def 2)	-0.10	-0.05	-0.05	0.10	-0.50	0.62		
Assigned to OIA	0.05	0.02	0.03	0.08	0.38	0.70	0.09	0.11
Assigned to control	-0.08	-0.02	-0.06	0.08	-0.78	0.43		

4 Breakdown of answers when careless and insufficient effort (C/IE) responses are defined as four identical values in the outcome measures

Table A8: Breakdown of answers when careless and insufficient effort (C/IE) responses are defined as four identical values in the outcome measures

Group	Total assigned	Total quality responses	Passed MC	Failed MC	Hard to say	Refused to answer	No engagement
East Slav identity affirmation (OIA)	675	617	279	137	121	7	73
Ukrainian identity affirmation (NIA)	663	605	277	158	104	10	56
Control (jellybeans)	662	616	320	32	69	5	190
Total	2,000	1,838					

5 Resultant samples and power of the test

Given the heterogeneity of responses to the manipulation check question, there are multiple ways to define which observations are considered "treated." First, the most restrictive conceptualization of "treated" includes only those respondents who passed the manipulation check—this conceptualization of treatment leaves 304 observations in the East Slav identity affirmation group, 301 respondents in the National Ukrainian affirmation group, and 334 control units. Throughout the paper, this most restrictive definition is labeled as 'definition 1.' After removing the C/IE responses of three consecutive values in the outcome measures, the resultant sample includes 267, 267, and 310 respondents in each group respectively (see 'definition 1' in Table A9). Based on the pre-registered design, the most restrictive sample allows sufficient power to uncover moderate effect size in the regression analysis, but not in t-tests, which is why we present only regression results for the restrictive sample. When 'careless/insufficient effort' (C/IE) responses are defined as four consecutive answers of the same value in outcome measures, the resultant groups include 279, 277, and 320 subjects respectively (see 'definition 1' in Table A10). These samples are insufficient for t-test analysis but allow enough power for regression analysis.

Table A9: Conceptualizations of treated when careless and insufficient effort (C/IE) responses are defined as three identical consecutive values in the outcome measures

Label	Treated defined as...	Sample size			Power sufficient for...		Analysis
		East Slav affirmation	Ukrainian affirmation	Control	t-test	OLS	shown in
Definition 1	Only those who passed MC	267	267	310	N	Y	Appendix
Definition 2	Passed MC + 'hard to say'	387	367	376	Y	Y	Paper
Assigned	Everyone assigned	598	582	590	Y	Y	Appendix

Table A10: Conceptualizations of treated when careless and insufficient effort (C/IE) responses are defined as four identical consecutive values in the outcome measures

Label	Treated defined as...	Sample size			Power sufficient for...		Analysis
		East Slav affirmation	Ukrainian affirmation	Control	t-test	OLS	shown in
Definition 1	Only those who passed MC	279	277	320	N	Y	Appendix
Definition 2	Passed MC + 'hard to say'	400	381	389	Y	Y	Appendix
Assigned	Everyone assigned	617	605	616	Y	Y	Appendix

Second, a less restrictive conceptualization of "treated" includes those respondents who passed the manipulation check or answered 'hard to say' when facing the manipulation check question (387, 367, 376 and 400, 381, 389 respondents in each group respectively, see Tables A9 and A10 for details). Hereafter, this conceptualization of "treated" is referred to as 'definition 2.' This definition allows us to obtain both differences-in-means and regression-based estimates.

Third, as an additional robustness check, we also conduct all analyses using only the indicator of whether a respondent was assigned to a given treatment regardless of whether they engaged with the interviewer during the exercise or the manipulation check questions (598, 582, 590 and

617, 605, 616 subjects respectively—'assigned'). This definition also provides sufficient sample size for the planned analyses.

Given high rates of failure to pass the manipulation check and high tendency of non-engagement with the manipulation check question, we also include a section—not planned in the preregistered design—that discusses the impact of Ukraine's regional heterogeneity on these rates.

6 Randomization: Are covariates balanced between treated and control groups?

6.1 Summary of randomization analyses

Table A11: Summary of randomization analyses

	**C/IE = 3 consecutive identical values**	**C/IE = 4 consecutive identical values**
OIA, definition 1	All balanced	Education: less educated individuals were more likely to pass the MC after the treatment than after the control.
OIA, definition 2	All balanced	Age, Education, Income, Retired: Older respondents and less educated individuals, and those with lower incomes, and those in retirement were more likely to pass the MC after treatment or to say "don't know."
Assigned to OIA	All balanced	All balanced
NIA, definition 1	Age, Russia Thermometer: Respondents with more positive views of Russia and older respondents were more likely to pass the MC after the treatment than after the control.	
NIA, definition 2	Age, Russia Thermometer: Respondents with more positive views of Russia and older respondents were more likely to pass the MC after treatment or to say "don't know."	
Assigned to NIA	Voted none: Respondents who support none of the political parties were more likely to be assigned to the NIA treatment.	

6.2 *Removing 'careless/insufficient effort' (C/IE) responses as* three *consecutive answers of the same value*

Table A12: Differences in observable attributes between those who passed the manipulation check after the Eastern Slav identity affirmation and those who passed the manipulation check after the control exercise (OIA, definition 1)

	OIA=0	OIA=1	Difference	t-statistic	p-value
Age	43.20	44.78	-1.58	-1.17	0.24
Female	1.59	1.53	0.06	1.50	0.13
Education	6.68	6.46	0.22	1.66	0.10
Income	2.69	2.61	0.08	1.15	0.25
Southeast	0.40	0.40	0.01	0.15	0.88
Thermometer twd Russia	-4.15	-2.72	-1.43	-0.46	0.64
Would vote for pro-Zelensky party	0.22	0.18	0.05	1.39	0.16
Would vote none	0.34	0.37	-0.03	-0.80	0.42
Professional	0.25	0.22	0.03	0.88	0.38
Retired	0.18	0.23	-0.05	-1.43	0.15
Settlement Type	4.73	4.49	0.24	1.27	0.21
Russian language	0.44	0.39	0.05	1.18	0.24
Russian ethnicity	0.06	0.04	0.02	1.15	0.25

Table A13: Differences in observable attributes between those who passed the manipulation check or answered “hard to say” after the Eastern Slav identity affirmation and those who passed the manipulation check or answered “hard to say” after the control exercise (OIA, definition 2)

	OIA=0	OIA=1	Difference	t-statistic	p-value
Age	44.47	46.51	-2.05	-1.72	0.09
Female	1.57	1.53	0.04	1.17	0.24
Education	6.63	6.43	0.20	1.70	0.09
Income	2.68	2.57	0.11	1.81	0.07
Southeast	0.40	0.40	0.01	0.25	0.80
Thermometer twd Russia	-3.44	-1.59	-1.85	-0.69	0.49
Would vote for pro-Zelensky party	0.21	0.18	0.03	1.20	0.23
Would vote none	0.34	0.37	-0.03	-0.84	0.40
Professional	0.23	0.21	0.02	0.82	0.41
Retired	0.20	0.26	-0.06	-2.01	0.04
Settlement Type	4.73	4.64	0.09	0.55	0.58
Russian language	0.42	0.41	0.01	0.34	0.74
Russian ethnicity	0.06	0.05	0.01	0.59	0.56

Table A14: Differences in observable attributes between those who assigned to the Eastern Slav identity affirmation treatment and those who were assigned to the control exercise (OIA, definition 3)

	OIA=0	OIA=1	Difference	t-statistic	p-value
Age	46.48	46.98	-0.51	-0.53	0.59
Female	1.54	1.53	0.02	0.60	0.55
Education	6.55	6.59	-0.04	-0.44	0.66
Income	2.61	2.61	-0.00	-0.02	0.99
Southeast	0.40	0.37	0.03	0.96	0.34
Thermometer twd Russia	-1.75	-1.52	-0.23	-0.10	0.92
Would vote for pro-Zelensky party	0.18	0.17	0.01	0.49	0.62
Would vote none	0.36	0.37	-0.01	-0.49	0.63
Professional	0.23	0.23	0.00	0.06	0.95
Retired	0.24	0.25	-0.01	-0.34	0.74
Settlement Type	4.75	4.85	-0.10	-0.77	0.44
Russian language	0.40	0.39	0.01	0.31	0.76
Russian ethnicity	0.05	0.05	0.00	0.32	0.75

Table A15: Differences in observable attributes between those who passed the manipulation check after the Ukrainian national identity affirmation and those who passed the manipulation check after the control exercise (NIA, definition 1)

	NIA=0	NIA=1	Difference	t-statistic	p-value
Age	43.20	46.27	-3.06	-2.31	0.02
Female	1.59	1.58	0.01	0.24	0.81
Education	6.68	6.68	-0.00	-0.01	0.99
Income	2.69	2.60	0.09	1.33	0.18
Southeast	0.40	0.39	0.01	0.34	0.74
Thermometer twd Russia	-4.15	2.40	-6.55	-2.11	0.04
Would vote for pro-Zelensky party	0.22	0.19	0.03	0.82	0.41
Would vote none	0.34	0.39	-0.05	-1.27	0.21
Professional	0.25	0.25	-0.01	-0.17	0.86
Retired	0.18	0.22	-0.04	-1.22	0.22
Settlement Type	4.73	4.63	0.10	0.49	0.62
Russian language	0.44	0.38	0.06	1.46	0.15
Russian ethnicity	0.06	0.05	0.01	0.29	0.77

Table A16: Differences in observable attributes between those who passed the manipulation check or answered "hard to say" after the Ukrainian national identity affirmation and those who passed the manipulation check or answered "hard to say" after the control exercise (NIA, definition 2)

	NIA=0	NIA=1	Difference	t-statistic	p-value
Age	44.47	47.61	-3.14	-2.66	0.01
Female	1.57	1.59	-0.02	-0.69	0.49
Education	6.63	6.58	0.04	0.40	0.69
Income	2.68	2.57	0.11	1.75	0.08
Southeast	0.40	0.40	0.01	0.25	0.80
Thermometer twd Russia	-3.44	2.48	-5.92	-2.14	0.03
Would vote for pro-Zelensky party	0.21	0.18	0.03	0.94	0.35
Would vote none	0.34	0.39	-0.05	-1.39	0.16
Professional	0.23	0.23	0.00	0.08	0.94
Retired	0.20	0.26	-0.06	-1.84	0.07
Settlement Type	4.73	4.61	0.12	0.71	0.48
Russian language	0.42	0.39	0.03	0.92	0.36
Russian ethnicity	0.06	0.06	-0.01	-0.39	0.69

Table A17: Differences in observable attributes between those who assigned to the Ukrainian national identity affirmation treatment and those who were assigned to the control exercise (NIA, definition 3)

	NIA=0	NIA=1	Difference	t-statistic	p-value
Age	46.48	47.86	-1.39	-1.46	0.14
Female	1.54	1.59	-0.05	-1.62	0.10
Education	6.55	6.63	-0.08	-0.83	0.41
Income	2.61	2.58	0.03	0.53	0.59
Southeast	0.40	0.43	-0.03	-1.20	0.23
Thermometer twd Russia	-1.75	1.85	-3.61	-1.61	0.11
Would vote for pro-Zelensky party	0.18	0.17	0.00	0.20	0.84
Would vote none	0.36	0.41	-0.05	-1.93	0.05
Professional	0.23	0.22	0.02	0.64	0.52
Retired	0.24	0.26	-0.02	-0.74	0.46
Settlement Type	4.75	4.80	-0.05	-0.36	0.72
Russian language	0.40	0.42	-0.01	-0.49	0.62
Russian ethnicity	0.05	0.07	-0.01	-0.93	0.35

6.3 Removing 'careless/insufficient effort' (C/IE) responses as four *consecutive answers of the same value*

Table A18: Differences in observable attributes between those who passed the manipulation check after the Eastern Slav identity affirmation and those who passed the manipulation check after the control exercise (OIA, definition 1; C/IE = 4 consecutive)

	OIA=0	OIA=1	Difference	t-statistic	p-value
Age	43.15	45.30	-2.15	-1.61	0.11
Female	1.58	1.54	0.04	1.06	0.29
Education	6.70	6.42	0.28	2.14	0.03
Income	2.70	2.58	0.12	1.76	0.08
Southeast	0.40	0.38	0.01	0.33	0.74
Thermometer twd Russia	-4.90	-3.28	-1.62	-0.54	0.59
Would vote for pro-Zelensky party	0.22	0.17	0.05	1.53	0.13
Would vote none	0.33	0.38	-0.05	-1.23	0.22
Professional	0.25	0.21	0.04	1.03	0.31
Retired	0.18	0.25	-0.06	-1.88	0.06
Settlement Type	4.73	4.47	0.26	1.36	0.17
Russian language	0.44	0.39	0.05	1.33	0.19
Russian ethnicity	0.06	0.04	0.02	1.18	0.24

Table A19: Differences in observable attributes between those who passed the manipulation check or answered "hard to say" after the Eastern Slav identity affirmation and those who passed the manipulation check or answered "hard to say" after the control exercise (OIA, definition 2; C/IE = 4 consecutive)

	OIA=0	OIA=1	Difference	t-statistic	p-value
Age	44.47	46.77	-2.30	-1.95	0.05
Female	1.56	1.53	0.03	0.79	0.43
Education	6.62	6.40	0.22	1.95	0.05
Income	2.69	2.55	0.14	2.19	0.03
Southeast	0.40	0.39	0.01	0.31	0.75
Thermometer twd Russia	-4.05	-2.02	-2.03	-0.77	0.44
Would vote for pro-Zelensky party	0.21	0.17	0.04	1.27	0.20
Would vote none	0.33	0.37	-0.04	-1.13	0.26
Professional	0.23	0.20	0.03	0.90	0.37
Retired	0.20	0.27	-0.07	-2.38	0.02
Settlement Type	4.70	4.62	0.09	0.54	0.59
Russian language	0.42	0.41	0.01	0.40	0.69
Russian ethnicity	0.05	0.04	0.01	0.58	0.56

Table A20: Differences in observable attributes between those who assigned to the Eastern Slav identity affirmation treatment and those who were assigned to the control exercise (OIA, definition 3; C/IE = 4 consecutive)

	OIA=0	OIA=1	Difference	t-statistic	p-value
Age	46.42	47.15	-0.73	-0.78	0.44
Female	1.54	1.53	0.01	0.43	0.67
Education	6.55	6.57	-0.02	-0.22	0.82
Income	2.62	2.60	0.02	0.32	0.75
Southeast	0.40	0.37	0.03	1.08	0.28
Thermometer twd Russia	-2.78	-2.07	-0.72	-0.33	0.74
Would vote for pro-Zelensky party	0.18	0.17	0.01	0.69	0.49
Would vote none	0.35	0.37	-0.02	-0.69	0.49
Professional	0.23	0.23	-0.00	-0.05	0.96
Retired	0.24	0.26	-0.01	-0.58	0.56
Settlement Type	4.75	4.83	-0.08	-0.66	0.51
Russian language	0.40	0.39	0.01	0.43	0.67
Russian ethnicity	0.05	0.05	0.00	0.40	0.69

Table A21: Differences in observable attributes between those who passed the manipulation check after the Ukrainian national identity affirmation and those who passed the manipulation check after the control exercise (NIA, definition 1; C/IE = 4 consecutive)

	NIA=0	NIA=1	Difference	t-statistic	p-value
Age	43.15	46.35	-3.20	-2.46	0.01
Female	1.58	1.57	0.02	0.43	0.67
Education	6.70	6.62	0.08	0.63	0.53
Income	2.70	2.60	0.10	1.46	0.15
Southeast	0.40	0.38	0.01	0.35	0.72
Thermometer twd Russia	-4.90	1.67	-6.57	-2.14	0.03
Would vote for pro-Zelensky party	0.22	0.20	0.02	0.59	0.56
Would vote none	0.33	0.38	-0.05	-1.30	0.19
Professional	0.25	0.25	-0.01	-0.16	0.87
Retired	0.18	0.22	-0.04	-1.09	0.28
Settlement Type	4.73	4.63	0.10	0.53	0.60
Russian language	0.44	0.38	0.06	1.52	0.13
Russian ethnicity	0.06	0.05	0.01	0.31	0.76

Table A22: Differences in observable attributes between those who passed the manipulation check or answered “hard to say” after the Ukrainian national identity affirmation and those who passed the manipulation check or answered “hard to say” after the control exercise (NIA, definition 2; C/IE = 4 consecutive)

	NIA=0	NIA=1	Difference	t-statistic	p-value
Age	44.47	47.52	-3.05	-2.62	0.01
Female	1.56	1.58	-0.02	-0.48	0.63
Education	6.62	6.54	0.09	0.80	0.43
Income	2.69	2.58	0.10	1.66	0.10
Southeast	0.40	0.39	0.01	0.21	0.83
Thermometer twd Russia	-4.05	1.63	-5.68	-2.08	0.04
Would vote for pro-Zelensky party	0.21	0.19	0.02	0.85	0.40
Would vote none	0.33	0.38	-0.05	-1.49	0.14
Professional	0.23	0.23	0.00	0.01	0.99
Retired	0.20	0.25	-0.05	-1.71	0.09
Settlement Type	4.70	4.61	0.09	0.54	0.59
Russian language	0.42	0.39	0.03	0.94	0.35
Russian ethnicity	0.05	0.06	-0.01	-0.38	0.70

Table A23: Differences in observable attributes between those who assigned to the Ukrainian national identity affirmation treatment and those who were assigned to the control exercise (NIA, definition 3; C/IE = 4 consecutive)

	NIA=0	NIA=1	Difference	t-statistic	p-value
Age	46.42	47.75	-1.33	-1.43	0.15
Female	1.54	1.58	-0.04	-1.28	0.20
Education	6.55	6.59	-0.04	-0.41	0.68
Income	2.62	2.59	0.03	0.49	0.62
Southeast	0.40	0.43	-0.03	-1.14	0.26
Thermometer twd Russia	-2.78	1.01	-3.79	-1.72	0.09
Would vote for pro-Zelensky party	0.18	0.18	0.00	0.15	0.88
Would vote none	0.35	0.41	-0.06	-2.25	0.02
Professional	0.23	0.22	0.01	0.45	0.65
Retired	0.24	0.26	-0.02	-0.64	0.52
Settlement Type	4.75	4.81	-0.06	-0.46	0.64
Russian language	0.40	0.41	-0.01	-0.32	0.75
Russian ethnicity	0.05	0.06	-0.01	-0.82	0.41

7 Analyses: T-tests

7.1 Effects of the OIA treatment on trust

7.1.1 Treatment conceptualized as definition 2: Those who passed the MC or said "hard to say"

Table A24: Differences in trust between those who passed the MC or said "hard to say" after the Eastern Slav identity affirmation treatment and those who passed the MC or said "hard to say" after the control exercise (OIA, definition 2; C/IE = 3 consecutive)

	OIA=0	OIA=1	Difference	t-statistic	p-value
Trust Russian Gov (2 items)	-0.00	0.04	-0.04	-0.63	0.53
Trust Russian Ppl (2 items)	0.18	0.20	-0.02	-0.34	0.73
Trust (4 items)	0.13	0.17	-0.03	-0.65	0.52

Table A25: Differences in trust between those who passed the MC or said "hard to say" after the Eastern Slav identity affirmation treatment and those who passed the MC or said "hard to say" after the control exercise (OIA, definition 2; C/IE = 4 consecutive)

	OIA=0	OIA=1	Difference	t-statistic	p-value
Trust Russian Gov (2 items)	-0.00	0.04	-0.04	-0.63	0.53
Trust Russian Ppl (2 items)	0.13	0.16	-0.03	-0.51	0.61
Trust (4 items)	0.10	0.14	-0.04	-0.70	0.48

7.1.2 Treatment conceptualized as everyone assigned to OIA

Table A26: Differences in trust between those assigned to the Eastern Slav identity affirmation treatment and those assigned to the control exercise (OIA, all assigned; C/IE = 3 consecutive)

	OIA=0	OIA=1	Difference	t-statistic	p-value
Trust Russian Gov (2 items)	-0.04	0.04	-0.08	-1.46	0.14
Trust Russian Ppl (2 items)	0.16	0.20	-0.03	-0.76	0.45
Trust (4 items)	0.10	0.17	-0.07	-1.62	0.11

Table A27: Differences in trust between those assigned to the Eastern Slav identity affirmation treatment and those assigned to the control exercise (OIA, all assigned; C/IE = 4 consecutive)

	OIA=0	OIA=1	Difference	t-statistic	p-value
Trust Russian Gov (2 items)	-0.04	0.04	-0.08	-1.46	0.14
Trust Russian Ppl (2 items)	0.11	0.16	-0.05	-1.14	0.26
Trust (4 items)	0.06	0.14	-0.08	-1.83	0.07

7.2 Effects of the NIA treatment on trust

7.2.1 The NIA treatment conceptualized as definition 2: Those who passed the MC or said "hard to say"

Table A28: Differences in trust between those who passed the MC or said "hard to say" after the Ukrainian national identity affirmation treatment and those who passed the MC or said "hard to say" after the control exercise (NIA, definition 2; C/IE = 3 consecutive)

	NIA=0	NIA=1	Difference	t-statistic	p-value
Trust Russian Gov (2 items)	-0.00	0.03	-0.03	-0.50	0.62
Trust Russian Ppl (2 items)	0.18	0.19	-0.02	-0.29	0.77
Trust (4 items)	0.13	0.16	-0.03	-0.51	0.61

Table A29: Differences in trust between those who passed the MC or said “hard to say” after the Ukrainian national identity affirmation treatment and those who passed the MC or said “hard to say” after the control exercise (NIA, definition 2; C/IE = 4 consecutive)

	NIA=0	NIA=1	Difference	t-statistic	p-value
Trust Russian Gov (2 items)	-0.00	0.03	-0.03	-0.50	0.62
Trust Russian Ppl (2 items)	0.13	0.15	-0.01	-0.24	0.81
Trust (4 items)	0.10	0.12	-0.02	-0.44	0.66

7.2.2 Treatment conceptualized as everyone assigned to NIA

Table A30: Differences in trust between those assigned to the Ukrainian national identity affirmation treatment and those assigned to the control exercise (NIA, all assigned; C/IE = 3 consecutive)

	NIA=0	NIA=1	Difference	t-statistic	p-value
Trust Russian Gov (2 items)	-0.04	0.06	-0.09	-1.70	0.09
Trust Russian Ppl (2 items)	0.16	0.19	-0.03	-0.73	0.47
Trust (4 items)	0.10	0.17	-0.07	-1.67	0.09

Table A31: Differences in trust between those assigned to the Ukrainian national identity affirmation treatment and those assigned to the control exercise (NIA, all assigned; C/IE = 4 consecutive)

	NIA=0	NIA=1	Difference	t-statistic	p-value
Trust Russian Gov (2 items)	-0.04	0.06	-0.09	-1.70	0.09
Trust Russian Ppl (2 items)	0.11	0.14	-0.03	-0.73	0.47
Trust (4 items)	0.06	0.13	-0.07	-1.63	0.10

8 Analysis: Regression

8.1 The impact of OIA

Table A32: The impact of overarching identity affirmation (OIA) on Ukrainians' trust toward Russia

	(1) Trust Gov β / SE	(2) Trust Ppl β / SE	(3) Trust β / SE	(4) Trust Gov β / SE	(5) Trust Ppl β / SE	(6) Trust β / SE	(7) Trust Gov β / SE	(8) Trust Ppl β / SE	(9) Trust β / SE
OIA treatment	0.0821	0.0396	0.0532						
(def 1)	(0.092)	(0.071)	(0.065)						
OIA treatment				0.0702	0.0281	0.0389			
(def 2)				(0.081)	(0.064)	(0.058)			
Assigned to OIA							0.109*	0.0428	0.0767
treatment							(0.065)	(0.052)	(0.048)
Female	0.125	0.0337	0.0830	0.110	0.0492	0.0849	0.113*	0.0402	0.0778*
	(0.102)	(0.075)	(0.071)	(0.086)	(0.067)	(0.062)	(0.064)	(0.053)	(0.047)
Age (in years)	-0.000675	-0.00532*	-0.00266	-0.00166	-0.00308	-0.00178	0.00125	-0.00100	0.000496
	(0.003)	(0.003)	(0.003)	(0.003)	(0.003)	(0.002)	(0.002)	(0.002)	(0.002)
Education	-0.0243	0.0324	0.00386	-0.0152	0.0415**	0.00810	-0.0112	0.0416**	0.0103
	(0.032)	(0.023)	(0.022)	(0.028)	(0.020)	(0.020)	(0.023)	(0.017)	(0.016)
Income	-0.119*	-0.0782*	-0.0970**	-0.144***	-0.0396	-0.0872**	-0.0715*	-0.0793**	-0.0683**
	(0.065)	(0.047)	(0.047)	(0.054)	(0.043)	(0.039)	(0.041)	(0.036)	(0.030)
Southeast	0.135	0.177**	0.167**	0.140	0.160**	0.167**	0.176**	0.108**	0.161***
	(0.100)	(0.073)	(0.073)	(0.088)	(0.066)	(0.066)	(0.070)	(0.054)	(0.053)
Thermometer	0.00906***	0.00702***	0.00810***	0.00883***	0.00742***	0.00826***	0.00896***	0.00653***	0.00781***
twd Russia	(0.001)	(0.001)	(0.001)	(0.001)	(0.001)	(0.001)	(0.001)	(0.001)	(0.001)
Would vote none	-0.214**	0.106	-0.0499	-0.248***	0.0749	-0.0912	-0.155**	0.0424	-0.0636
	(0.087)	(0.067)	(0.062)	(0.077)	(0.063)	(0.056)	(0.061)	(0.053)	(0.045)
Professional	0.00266	0.103	0.0509	0.0322	0.0923	0.0533	-0.0168	-0.00268	-0.00137
	(0.118)	(0.094)	(0.083)	(0.103)	(0.087)	(0.075)	(0.078)	(0.068)	(0.057)
Retired	-0.0826	0.167	0.0360	0.115	0.163	0.132	0.0359	0.0585	0.0202
	(0.146)	(0.126)	(0.108)	(0.139)	(0.108)	(0.097)	(0.110)	(0.087)	(0.078)
Settlement type	-0.00616	-0.00397	-0.00678	-0.0178	-0.0130	-0.0182	-0.0170	-0.0121	-0.0166*
	(0.020)	(0.016)	(0.015)	(0.018)	(0.014)	(0.013)	(0.013)	(0.011)	(0.010)
Russian language	0.262**	-0.0139	0.119	0.237**	0.0237	0.123	0.242***	0.125**	0.181***
	(0.130)	(0.078)	(0.089)	(0.109)	(0.069)	(0.075)	(0.080)	(0.055)	(0.056)
Russian ethnicity	0.521**	0.479***	0.505***	0.513***	0.347**	0.384***	0.547***	0.335***	0.428***
	(0.203)	(0.160)	(0.153)	(0.178)	(0.147)	(0.134)	(0.149)	(0.116)	(0.104)
Observations	526	528	534	679	681	693	1004	998	1025
R^2	0.251	0.200	0.296	0.250	0.186	0.288	0.247	0.168	0.285
AIC	1289.3	1129.4	1004.4	1694.2	1468.8	1339.0	2434.3	2121.2	1909.3

Note: OLS regressions coefficients are shown in cells and standard errors—in parentheses. $^{*}p < 0.1,^{**} p < 0.05,^{***} p < 0.01$. OIA treatment = overarching identity affirmation treatment indicates those respondents whose Eastern Slav identity was affirmed. Definition 1 defines treatment as as those who passed the manipulation check after the affirmation/control exercise; definition 2 defines treatment as those who passed the manipulation check or answered "hard to say" after the affirmation/control exercise; assigned to OIA treatment indicates everyone assigned regardless of whether they passed the manipulation check. Careless or insufficient effort (C/IE) responses are defined as **three** identical consecutive values in the outcome measures.

Table A33: The impact of overarching identity affirmation (OIA) on Ukrainians' trust toward Russia

	(1)<br>Trust Gov<br>β / SE	(2)<br>Trust Ppl<br>β / SE	(3)<br>Trust<br>β / SE	(4)<br>Trust Gov<br>β / SE	(5)<br>Trust Ppl<br>β / SE	(6)<br>Trust<br>β / SE	(7)<br>Trust Gov<br>β / SE	(8)<br>Trust Ppl<br>β / SE	(9)<br>Trust<br>β / SE
OIA treatment	0.0821	0.0240	0.0328						
(def 1)	(0.092)	(0.074)	(0.065)						
OIA treatment				0.0702	0.0165	0.0245			
(def 2)				(0.081)	(0.066)	(0.058)			
Assigned to OIA							0.109*	0.0392	0.0674
treatment							(0.065)	(0.055)	(0.048)
Female	0.125	0.00236	0.0589	0.110	0.0253	0.0646	0.113*	0.00648	0.0558
	(0.102)	(0.077)	(0.071)	(0.086)	(0.068)	(0.062)	(0.064)	(0.055)	(0.047)
Age (in years)	-0.000675	-0.00526*	-0.00291	-0.00166	-0.00365	-0.00263	0.00125	-0.00213	-0.000354
	(0.003)	(0.003)	(0.003)	(0.003)	(0.003)	(0.002)	(0.002)	(0.002)	(0.002)
Education	-0.0243	0.0369	0.00808	-0.0152	0.0522**	0.0177	-0.0112	0.0500***	0.0188
	(0.032)	(0.023)	(0.022)	(0.028)	(0.020)	(0.019)	(0.023)	(0.018)	(0.016)
Income	-0.119*	-0.0324	-0.0645	-0.144***	-0.0134	-0.0658*	-0.0715*	-0.0627*	-0.0558*
	(0.065)	(0.051)	(0.048)	(0.054)	(0.045)	(0.040)	(0.041)	(0.038)	(0.031)
Southeast	0.135	0.208***	0.191***	0.140	0.199***	0.200***	0.176**	0.121**	0.171***
	(0.100)	(0.074)	(0.073)	(0.088)	(0.067)	(0.066)	(0.070)	(0.055)	(0.053)
Thermometer	0.00906***	0.00710***	0.00817***	0.00883***	0.00735***	0.00822***	0.00896***	0.00681***	0.00794***
twd Russia	(0.001)	(0.001)	(0.001)	(0.001)	(0.001)	(0.001)	(0.001)	(0.001)	(0.001)
Would vote none	-0.214**	0.0513	-0.0816	-0.248***	0.0315	-0.122*	-0.155**	0.0152	-0.0824*
	(0.087)	(0.087)	(0.069)	(0.077)	(0.077)	(0.063)	(0.061)	(0.062)	(0.048)
Professional	0.00266	0.0817	0.0294	0.0322	0.0836	0.0432	-0.0168	-0.0111	-0.0136
	(0.118)	(0.096)	(0.084)	(0.103)	(0.088)	(0.076)	(0.078)	(0.069)	(0.057)
Retired	-0.0826	0.0797	-0.00758	0.115	0.132	0.128	0.0359	0.0692	0.0364
	(0.146)	(0.142)	(0.113)	(0.139)	(0.119)	(0.102)	(0.110)	(0.093)	(0.080)
Settlement Type	-0.00616	0.00361	-0.00263	-0.0178	-0.00430	-0.0122	-0.0170	-0.00758	-0.0138
	(0.020)	(0.017)	(0.015)	(0.018)	(0.015)	(0.013)	(0.013)	(0.012)	(0.010)
Russian language	0.262**	0.00892	0.130	0.237**	0.0541	0.143*	0.242***	0.155***	0.198***
	(0.130)	(0.078)	(0.088)	(0.109)	(0.069)	(0.075)	(0.080)	(0.056)	(0.056)
Russian ethnicity	0.521**	0.513***	0.532***	0.513***	0.374**	0.407***	0.547***	0.356***	0.441***
	(0.203)	(0.158)	(0.153)	(0.178)	(0.146)	(0.133)	(0.149)	(0.116)	(0.104)
Observations	526	549	555	679	706	718	1004	1039	1066
R^2	0.251	0.194	0.291	0.250	0.185	0.282	0.247	0.170	0.284
AIC	1289.3	1219.5	1061.0	1694.2	1572.1	1408.8	2434.3	2286.6	2012.1

Note: OLS regressions coefficients are shown in cells and standard errors—in parentheses. $^{*}p < 0.1,^{**} p < 0.05,^{***} p < 0.01$. OIA treatment = overarching identity affirmation treatment indicates those respondents whose Eastern Slav identity was affirmed. Definition 1 defines treatment as as those who passed the manipulation check after the affirmation/control exercise; definition 2 defines treatment as those who passed the manipulation check or answered "hard to say" after the affirmation/control exercise; assigned to OIA treatment indicates everyone assigned regardless of whether they passed the manipulation check. Careless or insufficient effort (C/IE) responses are defined as **four** identical consecutive values in the outcome measures.

Table A34: The impact of overarching identity affirmation (OIA) on Ukrainians' trust toward Russia; trust measured as raw items

	(1)<br>Trust Gov	(2)<br>Exploit	(3)<br>Trust Ppl	(4)<br>Kind	(5)<br>Trust Gov	(6)<br>Exploit	(7)<br>Trust Ppl	(8)<br>Kind
OIA treatment (def 1)	0.185	0.244	0.143	0.0108				
	(0.242)	(0.223)	(0.204)	(0.197)				
OIA treatment (def 2)					0.228	0.131	0.207	-0.0949
					(0.210)	(0.198)	(0.180)	(0.174)
Female	0.306	0.229	-0.0736	0.101	0.234	0.284	0.0549	0.129
	(0.260)	(0.247)	(0.206)	(0.199)	(0.219)	(0.208)	(0.182)	(0.177)
Age (in years)	-0.00609	-0.00101	-0.00911	-0.0125	-0.00720	-0.00355	-0.00447	-0.00757
	(0.010)	(0.008)	(0.008)	(0.009)	(0.009)	(0.008)	(0.007)	(0.007)
Education	0.0764	-0.117	0.0892	0.0350	0.0682	-0.0744	0.119*	0.0607
	(0.079)	(0.075)	(0.071)	(0.064)	(0.068)	(0.065)	(0.061)	(0.057)
Income	-0.118	-0.402**	-0.249*	-0.121	-0.231*	-0.333**	-0.116	-0.0776
	(0.170)	(0.162)	(0.147)	(0.131)	(0.140)	(0.133)	(0.127)	(0.116)
Southeast	0.359	0.252	0.402*	0.365*	0.330	0.292	0.330*	0.341*
	(0.258)	(0.244)	(0.222)	(0.211)	(0.222)	(0.214)	(0.193)	(0.188)
Thermometer twd Russia	0.0188***	0.0225***	0.0165***	0.0179***	0.0181***	0.0224***	0.0171***	0.0184***
	(0.004)	(0.004)	(0.004)	(0.003)	(0.003)	(0.003)	(0.003)	(0.003)
Would vote none	-0.529**	-0.287	0.382**	0.164	-0.628***	-0.312	0.218	0.163
	(0.243)	(0.231)	(0.191)	(0.196)	(0.219)	(0.198)	(0.174)	(0.179)
Professional	-0.252	0.127	0.515**	0.174	-0.103	0.0440	0.413*	0.0940
	(0.313)	(0.289)	(0.253)	(0.264)	(0.279)	(0.264)	(0.235)	(0.241)
Retired	0.183	-0.585	0.576	0.114	0.431	-0.151	0.499*	0.166
	(0.398)	(0.367)	(0.354)	(0.338)	(0.371)	(0.321)	(0.303)	(0.282)
Settlement Type	-0.0187	-0.00924	-0.0158	-0.0249	-0.0577	-0.0453	-0.0236	-0.0431
	(0.054)	(0.049)	(0.045)	(0.043)	(0.046)	(0.042)	(0.040)	(0.038)
Russian language	0.503*	0.546**	0.100	-0.102	0.453*	0.563***	0.168	-0.0533
	(0.274)	(0.255)	(0.221)	(0.221)	(0.233)	(0.214)	(0.195)	(0.192)
Russian ethnicity	0.788*	0.751**	1.258***	0.837*	0.917**	0.626**	0.987***	0.700*
	(0.459)	(0.362)	(0.390)	(0.484)	(0.388)	(0.312)	(0.375)	(0.390)
Observations	504	496	510	496	651	631	653	634
Pseudo R^2	0.087	0.105	0.081	0.055	0.091	0.103	0.072	0.054
AIC	1015.7	1183.2	1133.1	1433.6	1280.8	1450.9	1445.2	1764.5

Note: Ordered logistic regressions coefficients are shown in cells and standard errors—in parentheses. $^{*}p < 0.1,^{**} p < 0.05,^{***} p < 0.01$. OIA treatment = overarching identity affirmation treatment indicates those respondents whose Eastern Slav identity was affirmed. Definition 1 defines treatment as as those who passed the manipulation check after the affirmation/control exercise; definition 2 defines treatment as those who passed the manipulation check or answered "hard to say" after the affirmation/control exercise. Careless or insufficient effort (C/IE) responses are defined as **three** identical consecutive values in the outcome measures.

Table A35: The impact of overarching identity affirmation (OIA) on Ukrainians' trust toward Russia; trust measured as raw items

	(1) Trust Gov	(2) Exploit	(3) Trust Ppl	(4) Kind	(5) Trust Gov	(6) Exploit	(7) Trust Ppl	(8) Kind
OIA treatment (def 1)	0.124	0.180	0.0172	0.0681				
	(0.240)	(0.221)	(0.197)	(0.196)				
OIA treatment (def 2)					0.176	0.100	0.134	-0.0596
					(0.207)	(0.197)	(0.181)	(0.170)
Female	0.280	0.170	-0.153	0.0892	0.215	0.242	0.00566	0.111
	(0.260)	(0.247)	(0.195)	(0.197)	(0.218)	(0.209)	(0.174)	(0.175)
Age (in years)	-0.00803	-0.00247	-0.0104	-0.0100	-0.00978	-0.00726	-0.00936	-0.00573
	(0.010)	(0.008)	(0.008)	(0.008)	(0.009)	(0.008)	(0.008)	(0.007)
Education	0.0881	-0.102	0.0883	0.0331	0.0851	-0.0492	0.142***	0.0669
	(0.080)	(0.074)	(0.059)	(0.063)	(0.067)	(0.064)	(0.053)	(0.055)
Income	-0.0781	-0.320**	-0.0759	-0.113	-0.206	-0.277**	-0.0219	-0.0710
	(0.169)	(0.163)	(0.145)	(0.128)	(0.139)	(0.133)	(0.124)	(0.113)
Southeast	0.389	0.317	0.542***	0.317	0.383*	0.382*	0.495***	0.308*
	(0.258)	(0.246)	(0.206)	(0.211)	(0.222)	(0.215)	(0.181)	(0.187)
Thermometer twd Russia	0.0193***	0.0225***	0.0158***	0.0171***	0.0185***	0.0225***	0.0160***	0.0172***
	(0.004)	(0.004)	(0.003)	(0.003)	(0.003)	(0.003)	(0.003)	(0.003)
Would vote none	-0.576**	-0.334	0.251	0.141	-0.691***	-0.384*	0.0709	0.172
	(0.243)	(0.236)	(0.223)	(0.194)	(0.222)	(0.204)	(0.199)	(0.176)
Professional	-0.297	0.0521	0.382	0.178	-0.127	0.0166	0.358	0.103
	(0.311)	(0.296)	(0.264)	(0.254)	(0.279)	(0.269)	(0.239)	(0.231)
Retired	0.170	-0.654*	0.334	0.117	0.465	-0.0880	0.499	0.188
	(0.395)	(0.380)	(0.389)	(0.328)	(0.363)	(0.328)	(0.341)	(0.265)
Settlement Type	-0.0184	-0.00116	-0.00292	-0.0196	-0.0515	-0.0341	0.000311	-0.0401
	(0.054)	(0.049)	(0.045)	(0.042)	(0.046)	(0.042)	(0.039)	(0.038)
Russian language	0.491*	0.563**	0.116	-0.0470	0.467**	0.609***	0.225	-0.000414
	(0.274)	(0.254)	(0.205)	(0.222)	(0.234)	(0.213)	(0.182)	(0.191)
Russian ethnicity	0.841*	0.803**	1.302***	0.814*	0.956**	0.670**	1.026***	0.671*
	(0.464)	(0.359)	(0.374)	(0.484)	(0.388)	(0.309)	(0.360)	(0.391)
Observations	525	517	531	514	676	656	678	656
Pseudo R^2	0.090	0.104	0.073	0.050	0.093	0.103	0.070	0.048
AIC	1039.0	1214.3	1284.8	1493.1	1311.0	1489.6	1636.3	1860.1

Note: Ordered logistic regressions coefficients are shown in cells and standard errors—in parentheses. $^{*}p < 0.1, ^{**}p < 0.05, ^{***}p < 0.01$. OIA treatment = overarching identity affirmation treatment indicates those respondents whose Eastern Slav identity was affirmed. Definition 1 defines treatment as as those who passed the manipulation check after the affirmation/control exercise; definition 2 defines treatment as those who passed the manipulation check or answered "hard to say" after the affirmation/control exercise. Careless or insufficient effort (C/IE) responses are defined as **four** identical consecutive values in the outcome measures.

Table A36: The impact of overarching identity affirmation (OIA) on Ukrainians' trust toward Russia; trust measured as raw items

	(1) Trust Gov	(2) Exploit	(3) Trust Ppl	(4) Kind
Assigned to OIA treatment	0.298*	0.169	0.163	0.0181
	(0.176)	(0.165)	(0.151)	(0.150)
Female	0.307*	0.159	0.111	0.114
	(0.175)	(0.168)	(0.151)	(0.145)
Age (in years)	-0.000459	0.00154	0.000794	-0.000369
	(0.007)	(0.007)	(0.006)	(0.006)
Education	0.0500	-0.0471	0.116**	0.0568
	(0.061)	(0.056)	(0.054)	(0.048)
Income	-0.0477	-0.224**	-0.206*	-0.159
	(0.112)	(0.110)	(0.106)	(0.099)
Southeast	0.534***	0.218	0.222	0.198
	(0.193)	(0.177)	(0.162)	(0.158)
Thermometer twd Russia	0.0189***	0.0220***	0.0176***	0.0140***
	(0.003)	(0.003)	(0.003)	(0.002)
Would vote none	-0.379**	-0.204	0.127	0.0893
	(0.178)	(0.168)	(0.153)	(0.162)
Professional	-0.265	0.0480	0.0571	-0.0480
	(0.246)	(0.207)	(0.191)	(0.185)
Retired	0.168	-0.147	0.229	-0.126
	(0.282)	(0.274)	(0.262)	(0.240)
Settlement Type	-0.0413	-0.0566	-0.0158	-0.0440
	(0.039)	(0.035)	(0.032)	(0.031)
Russian language	0.448**	0.649***	0.308*	0.312**
	(0.193)	(0.174)	(0.159)	(0.159)
Russian ethnicity	0.910***	0.962***	0.840**	0.621**
	(0.320)	(0.268)	(0.331)	(0.302)
Observations	961	933	959	922
Pseudo R^2	0.092	0.098	0.070	0.040
AIC	1893.0	2148.7	2109.1	2608.5

Note: Ordered logistic regressions coefficients are shown in cells and standard errors—in parentheses. $^{*}p < 0.1, ^{**}p < 0.05, ^{***}p < 0.01$. OIA treatment = overarching identity affirmation treatment indicates those respondents whose Eastern Slav identity was affirmed. Assigned to OIA treatment indicates everyone assigned regardless of whether they passed the manipulation check. Careless or insufficient effort (C/IE) responses are defined as **three** identical consecutive values in the outcome measures.

Table A37: The impact of overarching identity affirmation (OIA) on Ukrainians' trust toward Russia; trust measured as raw items

	(1) Trust Gov	(2) Exploit	(3) Trust Ppl	(4) Kind
Assigned to OIA treatment	0.259	0.150	0.130	0.0318
	(0.175)	(0.165)	(0.152)	(0.147)
Female	0.275	0.111	0.0239	0.106
	(0.174)	(0.168)	(0.145)	(0.142)
Age (in years)	-0.00164	-0.00131	-0.00465	-0.000107
	(0.007)	(0.007)	(0.007)	(0.006)
Education	0.0680	-0.0226	0.136***	0.0605
	(0.060)	(0.055)	(0.049)	(0.047)
Income	-0.0348	-0.194*	-0.133	-0.153
	(0.111)	(0.110)	(0.104)	(0.095)
Southeast	0.551***	0.258	0.309**	0.173
	(0.192)	(0.176)	(0.153)	(0.156)
Thermometer twd Russia	0.0194***	0.0225***	0.0174***	0.0133***
	(0.003)	(0.003)	(0.002)	(0.002)
Would vote none	-0.415**	-0.253	0.0455	0.0880
	(0.179)	(0.171)	(0.165)	(0.158)
Professional	-0.299	0.0142	0.0653	-0.0570
	(0.243)	(0.208)	(0.188)	(0.179)
Retired	0.186	-0.0819	0.349	-0.0961
	(0.278)	(0.276)	(0.280)	(0.233)
Settlement Type	-0.0379	-0.0532	-0.00769	-0.0404
	(0.039)	(0.035)	(0.031)	(0.031)
Russian language	0.460**	0.693***	0.368**	0.342**
	(0.193)	(0.175)	(0.150)	(0.158)
Russian ethnicity	0.925***	0.990***	0.854***	0.612**
	(0.318)	(0.266)	(0.320)	(0.300)
Observations	1002	974	1000	956
Pseudo R^2	0.094	0.100	0.069	0.036
AIC	1935.3	2195.3	2378.4	2729.7

Note: Ordered logistic regressions coefficients are shown in cells and standard errors—in parentheses. $^{*}p < 0.1, ^{**}p < 0.05, ^{***}p < 0.01$. OIA treatment = overarching identity affirmation treatment indicates those respondents whose Eastern Slav identity was affirmed. Assigned to OIA treatment indicates everyone assigned regardless of whether they passed the manipulation check. Careless or insufficient effort (C/IE) responses are defined as **four** identical consecutive values in the outcome measures.

8.2 The impact of NIA

Table A38: The impact of national identity affirmation (NIA) on Ukrainians' trust toward Russia

	(1)<br>Trust Gov<br>β / SE	(2)<br>Trust Ppl<br>β / SE	(3)<br>Trust<br>β / SE	(4)<br>Trust Gov<br>β / SE	(5)<br>Trust Ppl<br>β / SE	(6)<br>Trust<br>β / SE	(7)<br>Trust Gov<br>β / SE	(8)<br>Trust Ppl<br>β / SE	(9)<br>Trust<br>β / SE
NIA treatment	0.0316	-0.00473	0.00846						
(def 1)	(0.084)	(0.071)	(0.061)						
NIA treatment				0.0402	0.0164	0.0250			
(def 2)				(0.075)	(0.064)	(0.055)			
Assigned to NIA							0.0655	-0.00101	0.0378
treatment							(0.064)	(0.054)	(0.049)
Female	0.134	0.0547	0.103	0.135*	0.0337	0.0884	0.0826	-0.0135	0.0285
	(0.092)	(0.077)	(0.069)	(0.081)	(0.069)	(0.061)	(0.065)	(0.056)	(0.049)
Age (in years)	0.00169	0.000833	0.00166	-0.000901	0.00312	0.00166	0.00169	0.00343	0.00341*
	(0.004)	(0.003)	(0.003)	(0.003)	(0.003)	(0.002)	(0.003)	(0.002)	(0.002)
Education	-0.0250	0.00364	-0.00794	-0.00805	0.0299	0.0148	0.0160	0.0363*	0.0287*
	(0.035)	(0.028)	(0.026)	(0.031)	(0.025)	(0.023)	(0.025)	(0.020)	(0.017)
Income	-0.0776	0.00739	-0.0303	-0.0708	0.0148	-0.0296	0.0106	-0.0213	-0.000714
	(0.058)	(0.046)	(0.044)	(0.048)	(0.043)	(0.038)	(0.042)	(0.036)	(0.032)
Southeast	0.0432	0.145*	0.107	0.0988	0.137**	0.128*	0.127*	0.137**	0.149***
	(0.106)	(0.074)	(0.075)	(0.093)	(0.067)	(0.065)	(0.073)	(0.055)	(0.052)
Thermometer	0.00710***	0.00600***	0.00647***	0.00800***	0.00682***	0.00738***	0.00863***	0.00657***	0.00753***
twd Russia	(0.001)	(0.001)	(0.001)	(0.001)	(0.001)	(0.001)	(0.001)	(0.001)	(0.001)
Would vote none	-0.128	0.0707	-0.0204	-0.163**	0.0490	-0.0444	-0.103	0.000458	-0.0451
	(0.090)	(0.076)	(0.067)	(0.080)	(0.070)	(0.061)	(0.065)	(0.056)	(0.048)
Professional	0.00594	0.0582	0.0286	-0.0524	0.0407	-0.0185	-0.0831	-0.0236	-0.0547
	(0.090)	(0.094)	(0.074)	(0.080)	(0.085)	(0.068)	(0.067)	(0.067)	(0.054)
Retired	0.00141	0.169	0.0853	0.116	0.107	0.0914	0.110	0.0178	0.0166
	(0.157)	(0.117)	(0.110)	(0.136)	(0.104)	(0.097)	(0.110)	(0.090)	(0.083)
Settlement type	-0.0132	-0.00824	-0.0103	-0.0125	-0.0223	-0.0168	-0.0111	-0.00711	-0.00956
	(0.020)	(0.015)	(0.014)	(0.018)	(0.014)	(0.012)	(0.014)	(0.011)	(0.010)
Russian language	0.326**	0.125	0.226**	0.287**	0.171**	0.228***	0.230***	0.134**	0.179***
	(0.139)	(0.080)	(0.091)	(0.124)	(0.072)	(0.080)	(0.087)	(0.059)	(0.058)
Russian ethnicity	0.538***	0.512***	0.519***	0.619***	0.614***	0.623***	0.604***	0.508***	0.560***
	(0.199)	(0.155)	(0.140)	(0.176)	(0.128)	(0.123)	(0.146)	(0.109)	(0.104)
Observations	516	516	525	656	654	669	977	976	998
R^2	0.213	0.179	0.258	0.249	0.220	0.306	0.239	0.194	0.288
AIC	1231.6	1107.2	985.1	1569.3	1406.6	1260.2	2348.4	2096.7	1881.5

Note: OLS regressions coefficients are shown in cells and standard errors—in parentheses. $^{*}p < 0.1,^{**} p < 0.05,^{***} p < 0.01$. NIA treatment = national identity affirmation treatment indicates those respondents whose Ukrainian national identity was affirmed. Definition 1 defines treatment as as those who passed the manipulation check after the affirmation/control exercise; definition 2 defines treatment as those who passed the manipulation check or answered "hard to say" after the affirmation/control exercise; assigned to NIA treatment indicates everyone assigned regardless of whether they passed the manipulation check. Careless or insufficient effort (C/IE) responses are defined as **three** identical consecutive values in the outcome measures.

Table A39: The impact of national identity affirmation (NIA) on Ukrainians' trust toward Russia

	(1) Trust Gov β / SE	(2) Trust Ppl β / SE	(3) Trust β / SE	(4) Trust Gov β / SE	(5) Trust Ppl β / SE	(6) Trust β / SE	(7) Trust Gov β / SE	(8) Trust Ppl β / SE	(9) Trust β / SE
NIA treatment (def 1)	0.0316 (0.084)	-0.00342 (0.072)	0.00622 (0.060)						
NIA treatment (def 2)				0.0402 (0.075)	0.0294 (0.064)	0.0351 (0.055)			
Assigned to NIA treatment							0.0655 (0.064)	0.00895 (0.055)	0.0430 (0.048)
Female	0.134 (0.092)	0.0850 (0.077)	0.119* (0.067)	0.135* (0.081)	0.0472 (0.069)	0.0925 (0.060)	0.0826 (0.065)	-0.0128 (0.057)	0.0276 (0.049)
Age (in years)	0.00169 (0.004)	-0.000287 (0.003)	0.00110 (0.003)	-0.000901 (0.003)	0.00112 (0.003)	0.000293 (0.003)	0.00169 (0.003)	0.00181 (0.002)	0.00251 (0.002)
Education	-0.0250 (0.035)	0.0106 (0.028)	-0.00112 (0.026)	-0.00805 (0.031)	0.0437* (0.025)	0.0267 (0.023)	0.0160 (0.025)	0.0444** (0.020)	0.0366** (0.017)
Income	-0.0776 (0.058)	-0.00316 (0.046)	-0.0378 (0.043)	-0.0708 (0.048)	0.0122 (0.043)	-0.0307 (0.037)	0.0106 (0.042)	-0.0225 (0.036)	-0.00243 (0.031)
Southeast	0.0432 (0.106)	0.169** (0.074)	0.121 (0.074)	0.0988 (0.093)	0.166** (0.067)	0.148** (0.065)	0.127* (0.073)	0.148*** (0.056)	0.155*** (0.051)
Thermometer twd Russia	0.00710*** (0.001)	0.00619*** (0.001)	0.00658*** (0.001)	0.00800*** (0.001)	0.00698*** (0.001)	0.00744*** (0.001)	0.00863*** (0.001)	0.00693*** (0.001)	0.00771*** (0.001)
Would vote none	-0.128 (0.090)	0.107 (0.076)	0.00262 (0.066)	-0.163** (0.080)	0.0616 (0.071)	-0.0406 (0.061)	-0.103 (0.065)	-0.000354 (0.057)	-0.0478 (0.049)
Professional	0.00594 (0.090)	0.0673 (0.092)	0.0283 (0.072)	-0.0524 (0.080)	0.0359 (0.084)	-0.0258 (0.066)	-0.0831 (0.067)	-0.0285 (0.067)	-0.0622 (0.053)
Retired	0.00141 (0.157)	0.197* (0.119)	0.101 (0.108)	0.116 (0.136)	0.165 (0.110)	0.134 (0.097)	0.110 (0.110)	0.0747 (0.093)	0.0500 (0.083)
Settlement Type	-0.0132 (0.020)	-0.0105 (0.016)	-0.0132 (0.014)	-0.0125 (0.018)	-0.0218 (0.014)	-0.0174 (0.012)	-0.0111 (0.014)	-0.0104 (0.011)	-0.0125 (0.010)
Russian language	0.326** (0.139)	0.119 (0.080)	0.220** (0.090)	0.287** (0.124)	0.158** (0.071)	0.218*** (0.079)	0.230*** (0.087)	0.142** (0.059)	0.183*** (0.057)
Russian ethnicity	0.538*** (0.199)	0.540*** (0.155)	0.541*** (0.138)	0.619*** (0.176)	0.653*** (0.129)	0.654*** (0.121)	0.604*** (0.146)	0.538*** (0.109)	0.580*** (0.103)
Observations	516	534	543	656	677	692	977	1018	1040
R^2	0.213	0.187	0.263	0.249	0.223	0.310	0.239	0.199	0.294
AIC	1231.6	1170.7	1023.8	1569.3	1484.8	1309.0	2348.4	2250.9	1973.2

Note: OLS regressions coefficients are shown in cells and standard errors—in parentheses. $^{*}p < 0.1,^{**} p < 0.05,^{***} p < 0.01$. NIA treatment = national identity affirmation treatment indicates those respondents whose Ukrainian national identity was affirmed. Definition 1 defines treatment as as those who passed the manipulation check after the affirmation/control exercise; definition 2 defines treatment as those who passed the manipulation check or answered "hard to say" after the affirmation/control exercise; assigned to NIA treatment indicates everyone assigned regardless of whether they passed the manipulation check. Careless or insufficient effort (C/IE) responses are defined as **four** identical consecutive values in the outcome measures.

Table A40: The impact of national identity affirmation (NIA) on Ukrainians' trust toward Russia; trust measured as raw items

	(1)<br>Trust Gov	(2)<br>Exploit	(3)<br>Trust Ppl	(4)<br>Kind	(5)<br>Trust Gov	(6)<br>Exploit	(7)<br>Trust Ppl	(8)<br>Kind
NIA treatment (def 1)	-0.0321	0.218	-0.0492	-0.105				
	(0.235)	(0.212)	(0.209)	(0.192)				
NIA treatment (def 2)					0.0382	0.159	0.0670	-0.0537
					(0.210)	(0.191)	(0.183)	(0.172)
Female	0.180	0.514**	0.109	0.0933	0.157	0.460**	0.113	0.0460
	(0.251)	(0.232)	(0.223)	(0.202)	(0.222)	(0.205)	(0.200)	(0.181)
Age (in years)	-0.00188	0.00241	-0.000888	0.0100	-0.00978	-0.00561	0.00315	0.0118*
	(0.012)	(0.008)	(0.009)	(0.008)	(0.011)	(0.008)	(0.008)	(0.007)
Education	-0.0496	-0.0748	-0.0275	0.0111	0.0127	-0.0324	0.0406	0.0703
	(0.092)	(0.078)	(0.080)	(0.082)	(0.082)	(0.069)	(0.072)	(0.072)
Income	-0.0899	-0.201	-0.00541	0.00173	-0.125	-0.190	-0.0115	0.0370
	(0.152)	(0.149)	(0.124)	(0.141)	(0.129)	(0.126)	(0.115)	(0.129)
Southeast	0.103	0.136	0.386	0.247	0.313	0.194	0.347	0.220
	(0.268)	(0.250)	(0.245)	(0.231)	(0.235)	(0.219)	(0.216)	(0.205)
Thermometer twd Russia	0.0170***	0.0165***	0.0165***	0.0156***	0.0187***	0.0176***	0.0176***	0.0162***
	(0.004)	(0.004)	(0.004)	(0.003)	(0.004)	(0.003)	(0.003)	(0.003)
Would vote none	-0.0926	-0.283	0.414*	-0.0954	-0.224	-0.381*	0.314	-0.145
	(0.255)	(0.245)	(0.234)	(0.223)	(0.232)	(0.215)	(0.212)	(0.200)
Professional	-0.00541	0.113	0.268	0.177	-0.127	-0.0251	0.142	0.164
	(0.250)	(0.254)	(0.272)	(0.247)	(0.240)	(0.230)	(0.248)	(0.227)
Retired	0.275	-0.0845	0.782**	-0.0461	0.426	0.189	0.608*	-0.104
	(0.431)	(0.372)	(0.319)	(0.311)	(0.381)	(0.321)	(0.313)	(0.281)
Settlement Type	-0.00383	-0.0545	-0.0291	-0.0500	-0.0311	-0.0329	-0.0467	-0.0782**
	(0.053)	(0.049)	(0.045)	(0.043)	(0.046)	(0.044)	(0.039)	(0.039)
Russian language	0.517*	0.751***	0.317	0.242	0.473*	0.620**	0.345	0.419**
	(0.282)	(0.274)	(0.235)	(0.223)	(0.249)	(0.251)	(0.210)	(0.201)
Russian ethnicity	0.819*	0.828**	1.256***	1.007**	1.155***	0.907***	1.551***	1.224***
	(0.492)	(0.393)	(0.432)	(0.430)	(0.435)	(0.334)	(0.359)	(0.355)
Observations	493	493	502	486	625	628	633	607
Pseudo R^2	0.071	0.079	0.085	0.050	0.089	0.082	0.093	0.060
AIC	946.1	1178.0	1086.2	1340.9	1143.0	1461.8	1367.5	1674.0

Note: Ordered logistic regressions coefficients are shown in cells and standard errors—in parentheses. $^{*}p < 0.1,^{**} p < 0.05,^{***} p < 0.01$. NIA treatment = national identity affirmation treatment indicates those respondents whose Ukrainian national identity was affirmed. Definition 1 defines treatment as as those who passed the manipulation check after the affirmation/control exercise; definition 2 defines treatment as those who passed the manipulation check or answered "hard to say" after the affirmation/control exercise. Careless or insufficient effort (C/IE) responses are defined as **three** identical consecutive values in the outcome measures.

Table A41: The impact of national identity affirmation (NIA) on Ukrainians' trust toward Russia; trust measured as raw items

	(1) Trust Gov	(2) Exploit	(3) Trust Ppl	(4) Kind	(5) Trust Gov	(6) Exploit	(7) Trust Ppl	(8) Kind
NIA treatment (def 1)	-0.0652	0.207	-0.0799	-0.0554				
	(0.233)	(0.211)	(0.203)	(0.189)				
NIA treatment (def 2)					0.0371	0.181	0.102	-0.0180
					(0.208)	(0.190)	(0.182)	(0.169)
Female	0.201	0.541**	0.201	0.0924	0.169	0.474**	0.177	0.0117
	(0.247)	(0.229)	(0.215)	(0.200)	(0.219)	(0.203)	(0.195)	(0.177)
Age (in years)	-0.00297	0.00168	-0.00346	0.00885	-0.0122	-0.00850	-0.00446	0.0105
	(0.012)	(0.008)	(0.009)	(0.008)	(0.011)	(0.007)	(0.009)	(0.007)
Education	-0.0365	-0.0564	0.00316	0.0213	0.0294	-0.00536	0.0948	0.0853
	(0.088)	(0.077)	(0.079)	(0.079)	(0.079)	(0.068)	(0.077)	(0.068)
Income	-0.116	-0.205	-0.0260	-0.00638	-0.139	-0.184	-0.0133	0.0431
	(0.151)	(0.148)	(0.120)	(0.137)	(0.127)	(0.125)	(0.113)	(0.124)
Southeast	0.107	0.169	0.456*	0.267	0.329	0.241	0.452**	0.239
	(0.266)	(0.249)	(0.237)	(0.227)	(0.233)	(0.218)	(0.209)	(0.202)
Thermometer twd Russia	0.0174***	0.0169***	0.0169***	0.0143***	0.0192***	0.0181***	0.0180***	0.0148***
	(0.004)	(0.004)	(0.003)	(0.003)	(0.004)	(0.003)	(0.003)	(0.003)
Would vote none	-0.0731	-0.224	0.524**	-0.0811	-0.241	-0.377*	0.291	-0.120
	(0.253)	(0.244)	(0.224)	(0.219)	(0.230)	(0.214)	(0.213)	(0.196)
Professional	-0.0108	0.105	0.254	0.186	-0.139	-0.0389	0.111	0.149
	(0.248)	(0.252)	(0.262)	(0.242)	(0.238)	(0.228)	(0.238)	(0.220)
Retired	0.329	-0.0416	0.878***	-0.0684	0.526	0.294	0.846**	-0.104
	(0.426)	(0.365)	(0.314)	(0.308)	(0.368)	(0.315)	(0.334)	(0.273)
Settlement Type	-0.0127	-0.0644	-0.0465	-0.0502	-0.0346	-0.0365	-0.0481	-0.0769**
	(0.053)	(0.049)	(0.044)	(0.043)	(0.046)	(0.043)	(0.038)	(0.038)
Russian language	0.491*	0.742***	0.290	0.285	0.444*	0.604**	0.284	0.438**
	(0.280)	(0.271)	(0.230)	(0.220)	(0.246)	(0.247)	(0.206)	(0.195)
Russian ethnicity	0.859*	0.858**	1.318***	1.015**	1.206***	0.963***	1.659***	1.228***
	(0.490)	(0.389)	(0.436)	(0.421)	(0.432)	(0.330)	(0.365)	(0.346)
Observations	511	511	520	501	648	651	656	627
Pseudo R^2	0.074	0.081	0.089	0.045	0.093	0.086	0.096	0.054
AIC	962.0	1193.2	1164.6	1383.4	1161.2	1481.7	1480.8	1744.4

Note: Ordered logistic regressions coefficients are shown in cells and standard errors—in parentheses. $^{*}p < 0.1, ^{**}p < 0.05, ^{***}p < 0.01$. NIA treatment = national identity affirmation treatment indicates those respondents whose Ukrainian national identity was affirmed. Definition 1 defines treatment as as those who passed the manipulation check after the affirmation/control exercise; definition 2 defines treatment as those who passed the manipulation check or answered "hard to say" after the affirmation/control exercise. Careless or insufficient effort (C/IE) responses are defined as **four** identical consecutive values in the outcome measures.

Table A42: The impact of national identity affirmation (NIA) on Ukrainians' trust toward Russia; trust measured as raw items

	(1) Trust Gov	(2) Exploit	(3) Trust Ppl	(4) Kind
Assigned to NIA treatment	0.0557	0.212	-0.0201	-0.0235
	(0.179)	(0.161)	(0.157)	(0.153)
Female	0.0891	0.246	-0.0791	-0.0232
	(0.183)	(0.168)	(0.162)	(0.150)
Age (in years)	-0.000824	-0.000361	0.00876	0.0111*
	(0.008)	(0.006)	(0.007)	(0.006)
Education	0.0400	0.0278	0.0981	0.0308
	(0.071)	(0.057)	(0.060)	(0.056)
Income	0.0633	-0.0195	-0.0744	-0.00387
	(0.112)	(0.104)	(0.096)	(0.100)
Southeast	0.502***	0.0777	0.331*	0.225
	(0.192)	(0.176)	(0.170)	(0.158)
Thermometer twd Russia	0.0192***	0.0191***	0.0182***	0.0133***
	(0.003)	(0.003)	(0.002)	(0.002)
Would vote none	-0.0913	-0.318*	0.168	-0.223
	(0.190)	(0.175)	(0.167)	(0.166)
Professional	-0.264	-0.0766	-0.0637	-0.0229
	(0.216)	(0.190)	(0.191)	(0.176)
Retired	0.231	0.177	0.238	-0.213
	(0.300)	(0.259)	(0.266)	(0.249)
Settlement Type	-0.0128	-0.0515	-0.0209	-0.0326
	(0.039)	(0.035)	(0.032)	(0.031)
Russian language	0.308	0.677***	0.212	0.407**
	(0.204)	(0.199)	(0.174)	(0.165)
Russian ethnicity	1.070***	0.948***	1.368***	1.026***
	(0.325)	(0.263)	(0.317)	(0.286)
Observations	929	928	947	905
Pseudo R^2	0.085	0.082	0.082	0.047
AIC	1781.4	2213.8	2125.4	2582.1

Note: Ordered logistic regressions coefficients are shown in cells and standard errors—in parentheses. $^{*}p < 0.1, ^{**}p < 0.05, ^{***}p < 0.01$. NIA treatment = national identity affirmation treatment indicates those respondents whose Ukrainian national identity was affirmed. Assigned to NIA treatment indicates everyone assigned regardless of whether they passed the manipulation check. Careless or insufficient effort (C/IE) responses are defined as **three** identical consecutive values in the outcome measures.

Table A43: The impact of national identity affirmation (NIA) on Ukrainians' trust toward Russia; trust measured as raw items

	(1)<br>Trust Gov	(2)<br>Exploit	(3)<br>Trust Ppl	(4)<br>Kind
Assigned to NIA treatment	0.0626	0.226	0.0194	-0.0281
	(0.176)	(0.160)	(0.151)	(0.149)
Female	0.0725	0.252	-0.0430	-0.0293
	(0.180)	(0.167)	(0.155)	(0.146)
Age (in years)	-0.00133	-0.00196	0.00339	0.00970*
	(0.008)	(0.006)	(0.007)	(0.006)
Education	0.0551	0.0463	0.128**	0.0403
	(0.069)	(0.056)	(0.059)	(0.053)
Income	0.0560	-0.0182	-0.0686	-0.00806
	(0.111)	(0.104)	(0.093)	(0.097)
Southeast	0.504***	0.0962	0.384**	0.221
	(0.190)	(0.175)	(0.164)	(0.155)
Thermometer twd Russia	0.0197***	0.0199***	0.0189***	0.0123***
	(0.003)	(0.003)	(0.002)	(0.002)
Would vote none	-0.0971	-0.321*	0.150	-0.220
	(0.188)	(0.175)	(0.165)	(0.161)
Professional	-0.269	-0.0945	-0.0614	-0.0412
	(0.213)	(0.188)	(0.183)	(0.172)
Retired	0.269	0.248	0.445	-0.201
	(0.293)	(0.256)	(0.278)	(0.244)
Settlement Type	-0.0168	-0.0602*	-0.0353	-0.0330
	(0.039)	(0.035)	(0.030)	(0.031)
Russian language	0.308	0.686***	0.230	0.417***
	(0.201)	(0.198)	(0.167)	(0.161)
Russian ethnicity	1.085***	0.986***	1.408***	1.040***
	(0.321)	(0.261)	(0.314)	(0.278)
Observations	969	970	989	940
Pseudo R^2	0.088	0.087	0.085	0.043
AIC	1817.5	2246.6	2318.7	2689.6

Note: Ordered logistic regressions coefficients are shown in cells and standard errors—in parentheses. $^{*}p < 0.1, ^{**}p < 0.05, ^{***}p < 0.01$. NIA treatment = national identity affirmation treatment indicates those respondents whose Ukrainian national identity was affirmed. Assigned to NIA treatment indicates everyone assigned regardless of whether they passed the manipulation check. Careless or insufficient effort (C/IE) responses are defined as **four** identical consecutive values in the outcome measures.

9 Analysis: Why were some respondents more likely to answer 'Hard to say' to trust questions?

This section replicates main results modeling "don't know" responses as a separate answer category. The KIIS uses "Hard to say" as the label for "Don't know" response.

We examine whether controls or treatments are associated with respondents' tendency to say 'Hard to say' when answering outcome questions about trusting Russia. This section uses binary indicators of whether a respondent answered 'hard to say' when facing the outcome questions on trust.

Our main finding is that older individuals and those who reported that they would support none of the political parties tended to answer 'Hard to say' to trust questions.

Table A44: Descriptive statistics for dichotomous dependent variables where answer 'Hard to say' is coded as '1'

	count	mean	sd	min	max	sum
Hard to say =1 (Trust Gov)	1770	.0570621	.2320269	0	1	101
Hard to say =1 (Trust Ppl)	1770	.0706215	.2562639	0	1	125
Hard to say =1 (Overall Trust)	1770	.0305085	.1720303	0	1	54
Observations	1770					

Note: Careless or insufficient effort (C/IE) responses are defined as **three** identical consecutive values in the outcome measures.

Table A45: Descriptive statistics for dichotomous dependent variables where answer 'Hard to say' is coded as '1'

	count	mean	sd	min	max	sum
Hard to say =1 (Trust Gov)	1838	.0919478	.2890308	0	1	169
Hard to say =1 (Trust Ppl)	1838	.0680087	.2518294	0	1	125
Hard to say =1 (Overall Trust)	1838	.0293798	.1689145	0	1	54
Observations	1838					

Note: Careless or insufficient effort (C/IE) responses are defined as **four** identical consecutive values in the outcome measures.

Table A46: The impact of socio-economic covariates on respondents' tendency to answer 'hard to say' when answering trust questions

	(1)	(2)	(3)	(4)	(5)	(6)
	Trust Gov	Trust Ppl	Overall Trust	Trust Gov	Trust Ppl	Overall Trust
	C/IE = 3 consecutive values			C/IE = 4 consecutive values		
Female	0.0613	-0.0249	0.718	0.0991	-0.0540	0.696
	(0.427)	(0.371)	(0.671)	(0.253)	(0.371)	(0.670)
Age (in years)	0.0601***	0.0143	0.0417*	0.0417***	0.0125	0.0401*
	(0.015)	(0.012)	(0.022)	(0.011)	(0.011)	(0.022)
Education	0.109	0.124	0.161	-0.0332	0.141	0.173
	(0.158)	(0.146)	(0.218)	(0.100)	(0.145)	(0.214)
Income	0.0576	-0.159	0.242	-0.0645	-0.131	0.275
	(0.229)	(0.287)	(0.414)	(0.158)	(0.289)	(0.420)
Southeast	0.778**	0.683	0.981*	0.0575	0.724*	1.023*
	(0.375)	(0.417)	(0.562)	(0.240)	(0.414)	(0.556)
Thermometer twd Russia	0.0168***	0.00432	0.00956	0.000534	0.00497	0.0101
	(0.005)	(0.005)	(0.006)	(0.003)	(0.005)	(0.006)
Would vote none	0.927***	0.899***	1.138**	0.459*	0.862***	1.104**
	(0.331)	(0.326)	(0.491)	(0.263)	(0.329)	(0.488)
Professional	-0.710	0.324	-1.860*	-0.215	0.293	-1.876*
	(0.510)	(0.530)	(1.068)	(0.343)	(0.529)	(1.056)
Retired	-0.779	0.757	0.747	-0.699*	0.804*	0.789
	(0.544)	(0.483)	(0.658)	(0.403)	(0.474)	(0.632)
Settlement Type	-0.0844	-0.141	-0.351*	-0.0283	-0.141	-0.350*
	(0.116)	(0.101)	(0.188)	(0.071)	(0.101)	(0.186)
Russian language	0.334	-0.236	0.726	-0.0420	-0.222	0.750
	(0.391)	(0.415)	(0.554)	(0.287)	(0.416)	(0.553)
Russian ethnicity	-0.00436	-0.137	0.391	-0.0450	-0.119	0.380
	(0.765)	(0.885)	(1.335)	(0.599)	(0.891)	(1.336)
Observations	1537	1537	1537	1596	1596	1596
Pseudo R^2	0.147	0.075	0.216	0.045	0.075	0.218
AIC	452.2	583.0	247.8	868.1	589.2	249.3

Note: Logistic regressions. $^{*}p < 0.1, ^{**}p < 0.05, ^{***}p < 0.01$. Dependent measures are dichotomous indicators of whether a respondent answered 'Hard to say' when answering trust questions.

Table A47: The impact of treatments (definition 1) and socio-economic covariates on respondents' tendency to answer 'hard to say' when answering trust questions

	(1)<br>Trust Gov	(2)<br>Trust Ppl	(3)<br>Overall Trust	(4)<br>Trust Gov	(5)<br>Trust Ppl	(6)<br>Overall Trust
OIA treatment (def 1)	0.272	-0.0111	1.406			
	(0.726)	(0.640)	(1.445)			
NIA treatment (def 1)				0.795	1.197*	2.174*
				(0.552)	(0.619)	(1.242)
Female	0.130	-0.777	0	0.198	0.537	-0.00398
	(0.673)	(0.675)	(.)	(0.793)	(0.785)	(1.433)
Age (in years)	0.0720***	0.0259	0.0104	0.0477*	0.0500*	0.0291
	(0.021)	(0.031)	(0.039)	(0.026)	(0.028)	(0.043)
Education	-0.128	0.111	0.355	0.171	0.0916	0.346
	(0.191)	(0.115)	(0.221)	(0.223)	(0.191)	(0.406)
Income	0.670	0.0508	1.084	-0.430	0.102	-0.264
	(0.437)	(0.765)	(0.921)	(0.418)	(0.459)	(0.774)
Southeast	1.004	2.300**	0	0.286	0.489	1.055*
	(1.015)	(1.052)	(.)	(0.821)	(0.494)	(0.642)
Thermometer twd Russia	0.0315***	0.00685	0.00905	0.0168*	0.00909	0.0176
	(0.008)	(0.006)	(0.010)	(0.010)	(0.008)	(0.017)
Would vote none	1.592*	0.926	3.335*	1.116	1.039	1.585
	(0.819)	(1.042)	(1.736)	(0.739)	(0.726)	(1.205)
Professional	-0.568	0.111	0	-1.161	-1.308	0
	(1.060)	(1.141)	(.)	(1.209)	(1.096)	(.)
Retired	-1.266	0.803	1.854	-0.687	0.512	1.515
	(1.259)	(1.021)	(1.666)	(1.163)	(1.029)	(2.217)
Settlement Type	0.248*	-0.0407	0.184	0.0799	0.0771	0.0145
	(0.141)	(0.166)	(0.137)	(0.118)	(0.126)	(0.246)
Russian language	0.720	-0.291	-1.881	0.0908	-0.163	-0.0193
	(1.006)	(0.901)	(1.238)	(0.642)	(0.578)	(0.565)
Russian ethnicity	0	0	0	0	0	0
	(.)	(.)	(.)	(.)	(.)	(.)
Observations	508	508	70	500	500	376
Pseudo R^2	0.250	0.159	0.415	0.163	0.181	0.280
AIC	96.84	78.59	31.39	133.0	111.3	59.50

Note: Logistic regressions. $^{*}p < 0.1,^{**}p < 0.05,^{***}p < 0.01$. Dependent measures are dichotomous indicators of whether a respondent answered 'Hard to say' when answering trust questions. Careless or insufficient effort (C/IE) responses are defined as **three** identical consecutive values in the outcome measures. A coefficient estimate of 0 and missing standard error indicate situations where the variable was removed due to perfect separation.

Table A48: The impact of treatments (definition 2) and socio-economic covariates on respondents' tendency to answer 'hard to say' when answering trust questions

	(1) Trust Gov	(2) Trust Ppl	(3) Overall Trust	(4) Trust Gov	(5) Trust Ppl	(6) Overall Trust
OIA treatment (def 2)	0.849	0.277	2.393			
	(0.607)	(0.555)	(2.140)			
NIA treatment (def 2)				0.785	0.992*	2.050*
				(0.515)	(0.511)	(1.177)
Female	-0.0921	-1.664***	0	-0.211	0.0901	-0.143
	(0.545)	(0.639)	(.)	(0.656)	(0.608)	(1.490)
Age (in years)	0.0816***	0.0476**	0.0456	0.0562**	0.0362**	0.0241
	(0.020)	(0.019)	(0.036)	(0.022)	(0.018)	(0.038)
Education	0.113	-0.127	-0.167	0.252	-0.0281	0.354
	(0.173)	(0.177)	(0.369)	(0.195)	(0.203)	(0.479)
Income	0.582*	-0.288	1.120	-0.781**	-0.115	-0.332
	(0.342)	(0.533)	(0.963)	(0.388)	(0.422)	(0.851)
Southeast	1.018	1.562**	3.661**	0.370	0.499	0.876
	(0.713)	(0.624)	(1.530)	(0.729)	(0.471)	(0.730)
Thermometer twd Russia	0.0319***	0.00443	0.0167	0.0215**	0.00287	0.0154
	(0.006)	(0.007)	(0.019)	(0.010)	(0.007)	(0.019)
Would vote none	1.581***	0.910	2.587*	1.351**	0.999*	1.488
	(0.553)	(0.708)	(1.481)	(0.606)	(0.565)	(1.323)
Professional	-0.546	1.054*	0.425	-0.376	-0.404	0
	(0.828)	(0.629)	(1.792)	(0.748)	(0.692)	(.)
Retired	-1.667**	-0.494	0.998	-1.406	-0.0691	1.298
	(0.842)	(0.784)	(1.827)	(1.016)	(0.857)	(2.257)
Settlement Type	0.337**	0.157	0.219**	0.0430	0.0974	-0.0309
	(0.139)	(0.107)	(0.090)	(0.113)	(0.121)	(0.298)
Russian language	0.156	-0.859	-0.944	-0.408	-0.147	0.0319
	(0.612)	(0.587)	(0.653)	(0.636)	(0.486)	(0.640)
Russian ethnicity	-0.0232	0	0	-1.057	0	0
	(0.861)	(.)	(.)	(0.982)	(.)	(.)
Observations	697	658	304	673	632	483
Pseudo R^2	0.270	0.171	0.361	0.191	0.098	0.233
AIC	138.2	119.8	44.97	166.3	154.2	63.45

Note: Logistic regressions. $^{*}p < 0.1, ^{**}p < 0.05, ^{***}p < 0.01$. Dependent measures are dichotomous indicators of whether a respondent answered 'Hard to say' when answering trust questions. Careless or insufficient effort (C/IE) responses are defined as **three** identical consecutive values in the outcome measures. A coefficient estimate of 0 and missing standard error indicate situations where the variable was removed due to perfect separation.

Table A49: The impact of being assigned to a certain treatment and socio-economic covariates on respondents' tendency to answer 'hard to say' when answering trust questions

	(1) Trust Gov	(2) Trust Ppl	(3) Overall Trust	(4) Trust Gov	(5) Trust Ppl	(6) Overall Trust
Assigned to OIA treatment	-0.0652	-0.0175	-0.544			
	(0.459)	(0.442)	(0.835)			
Assigned to NIA treatment				0.722*	0.0537	0.830
				(0.430)	(0.475)	(0.676)
Female	-0.0789	-0.397	-0.262	0.334	0.190	1.046
	(0.478)	(0.419)	(0.888)	(0.494)	(0.440)	(0.897)
Age (in years)	0.0794***	0.0142	0.0776***	0.0566***	0.00784	0.0415
	(0.016)	(0.014)	(0.024)	(0.018)	(0.017)	(0.027)
Education	-0.151	0.00153	-0.223	0.0553	0.129	0.0341
	(0.144)	(0.132)	(0.190)	(0.169)	(0.158)	(0.229)
Income	0.523	-0.0256	0.923	-0.140	-0.0583	0.0341
	(0.347)	(0.391)	(0.600)	(0.296)	(0.332)	(0.564)
Southeast	0.473	0.513	0.403	0.579	0.872	0.757
	(0.433)	(0.518)	(0.746)	(0.428)	(0.537)	(0.516)
Thermometer twd Russia	0.0197***	0.00275	0.00721	0.0144***	0.00909	0.00601
	(0.004)	(0.006)	(0.005)	(0.006)	(0.006)	(0.007)
Would vote none	0.859*	0.977**	0.903	0.947**	0.684*	1.106*
	(0.441)	(0.453)	(0.801)	(0.374)	(0.369)	(0.610)
Professional	-0.305	0.675	-0.528	-0.719	0.259	0
	(0.582)	(0.556)	(0.885)	(0.602)	(0.672)	(.)
Retired	-1.184*	0.583	-0.0681	-0.763	1.177*	0.542
	(0.627)	(0.576)	(0.885)	(0.611)	(0.639)	(0.682)
Settlement Type	0.162	-0.0570	-0.00217	-0.131	-0.237**	-0.407**
	(0.113)	(0.099)	(0.156)	(0.119)	(0.113)	(0.205)
Russian language	0.414	-0.790*	0.0887	0.555	0.0310	1.299**
	(0.400)	(0.422)	(0.657)	(0.470)	(0.495)	(0.579)
Russian ethnicity	-0.0821	-0.208	-0.0944	-0.614	-0.547	0.294
	(0.693)	(0.846)	(1.221)	(1.132)	(1.276)	(1.648)
Observations	1036	1036	1036	1011	1011	778
Pseudo R^2	0.179	0.064	0.155	0.158	0.121	0.239
AIC	240.2	391.4	125.7	338.8	386.8	192.4

Note: Logistic regressions. $^{*}p < 0.1, ^{**}p < 0.05, ^{***}p < 0.01$. Dependent measures are dichotomous indicators of whether a respondent answered 'Hard to say' when answering trust questions. Careless or insufficient effort (C/IE) responses are defined as **three** identical consecutive values in the outcome measures. A coefficient estimate of 0 and missing standard error indicate situations where the variable was removed due to perfect separation.

10 Analysis: Understanding the results through regional heterogeneity in Ukraine

10.1 Region-level effects of the OIA treatment on trust

10.1.1 The OIA treatment conceptualized based on definition 2: Those who passed the MC or said "hard to say"

Table A50: Differences in trust between those who passed the MC or said "hard to say" after the Eastern Slav identity affirmation treatment/the control exercise (OIA, definition 2; C/IE = 4 consecutive)

	OIA=0	OIA=1	Difference	t-statistic	p-value
Trust Russian Gov (2 items, full sample)	-0.00	0.04	-0.04	-0.63	0.53
Trust Russian Gov (2 items, West-Center)	-0.12	-0.11	-0.01	-0.10	0.92
Trust Russian Gov (2 items, Southeast)	0.17	0.30	-0.13	-1.03	0.30
Trust Russian Ppl (2 items, full sample)	0.13	0.16	-0.03	-0.51	0.61
Trust Russian Ppl (2 items, West-Center)	0.01	0.03	-0.03	-0.38	0.70
Trust Russian Ppl (2 items, Southeast)	0.33	0.38	-0.05	-0.51	0.61
Trust (4 items, full sample)	0.10	0.14	-0.04	-0.70	0.48
Trust (4 items, West-Center)	-0.02	-0.02	-0.00	-0.02	0.98
Trust (4 items, Southeast)	0.29	0.39	-0.11	-1.12	0.26

10.1.2 *Treatment conceptualized as everyone assigned to OIA*

Table A51: Differences in trust between those assigned to the Eastern Slav identity affirmation treatment and those assigned to the control exercise (OIA, all assigned; C/IE = 3 consecutive)

	OIA=0	OIA=1	Difference	t-statistic	p-value
Trust Russian Gov (2 items, full sample)	-0.04	0.04	-0.08	-1.46	0.14
Trust Russian Gov (2 items, West-Center)	-0.16	-0.10	-0.06	-0.94	0.35
Trust Russian Gov (2 items, Southeast)	0.14	0.30	-0.16	-1.60	0.11
Trust Russian Ppl (2 items, full sample)	0.16	0.20	-0.03	-0.76	0.45
Trust Russian Ppl (2 items, West-Center)	0.05	0.08	-0.03	-0.49	0.63
Trust Russian Ppl (2 items, Southeast)	0.32	0.38	-0.07	-0.89	0.37
Trust (4 items, full sample)	0.10	0.17	-0.07	-1.62	0.11
Trust (4 items, West-Center)	-0.01	0.03	-0.05	-0.98	0.33
Trust (4 items, Southeast)	0.26	0.39	-0.13	-1.74	0.08

Table A52: Differences in trust between those assigned to the Eastern Slav identity affirmation treatment and those assigned to the control exercise (OIA, all assigned; C/IE = 4 consecutive)

	OIA=0	OIA=1	Difference	t-statistic	p-value
Trust Russian Gov (2 items, full sample)	-0.04	0.04	-0.08	-1.46	0.14
Trust Russian Gov (2 items, West-Center)	-0.16	-0.10	-0.06	-0.94	0.35
Trust Russian Gov (2 items, Southeast)	0.14	0.30	-0.16	-1.60	0.11
Trust Russian Ppl (2 items, full sample)	0.11	0.16	-0.05	-1.14	0.26
Trust Russian Ppl (2 items, West-Center)	0.00	0.04	-0.04	-0.73	0.47
Trust Russian Ppl (2 items, Southeast)	0.27	0.36	-0.09	-1.24	0.22
Trust (4 items, full sample)	0.06	0.14	-0.08	-1.83	0.07
Trust (4 items, West-Center)	-0.05	0.00	-0.05	-1.09	0.27
Trust (4 items, Southeast)	0.23	0.38	-0.15	-1.98	0.05

10.2 Region-level effects of the NIA treatment on trust

10.2.1 The NIA treatment conceptualized based on definition 2: Those who passed the MC or said "hard to say"

Table A53: Differences in trust between those who passed the MC or said "hard to say" after the Ukrainian national identity affirmation treatment and those who were assigned to the control exercise (NIA, definition 2; C/IE = 4 consecutive)

	NIA=0	NIA=1	Difference	t-statistic	p-value
Trust Russian Gov (2 items, full sample)	-0.00	0.03	-0.03	-0.50	0.62
Trust Russian Gov (2 items, West-Center)	-0.12	-0.24	0.12	1.73	0.08
Trust Russian Gov (2 items, Southeast)	0.17	0.46	-0.29	-2.40	0.02
Trust Russian Ppl (2 items, full sample)	0.13	0.15	-0.01	-0.24	0.81
Trust Russian Ppl (2 items, West-Center)	0.01	-0.04	0.04	0.61	0.54
Trust Russian Ppl (2 items, Southeast)	0.33	0.45	-0.12	-1.15	0.25
Trust (4 items, full sample)	0.10	0.12	-0.02	-0.44	0.66
Trust (4 items, West-Center)	-0.02	-0.11	0.09	1.63	0.10
Trust (4 items, Southeast)	0.29	0.49	-0.21	-2.18	0.03

10.2.2 Treatment conceptualized as everyone assigned to NIA

Table A54: Differences in trust between those assigned to the Ukrainian national identity affirmation treatment and those assigned to the control exercise (NIA, all assigned; C/IE = 3 consecutive)

	NIA=0	NIA=1	Difference	t-statistic	p-value
Trust Russian Gov (2 items, full sample)	-0.04	0.06	-0.09	-1.70	0.09
Trust Russian Gov (2 items, West-Center)	-0.16	-0.17	0.02	0.32	0.75
Trust Russian Gov (2 items, Southeast)	0.14	0.37	-0.23	-2.42	0.02
Trust Russian Ppl (2 items, full sample)	0.16	0.19	-0.03	-0.73	0.47
Trust Russian Ppl (2 items, West-Center)	0.05	0.03	0.03	0.46	0.65
Trust Russian Ppl (2 items, Southeast)	0.32	0.42	-0.10	-1.34	0.18
Trust (4 items, full sample)	0.10	0.17	-0.07	-1.67	0.09
Trust (4 items, West-Center)	-0.01	-0.04	0.02	0.43	0.67
Trust (4 items, Southeast)	0.26	0.44	-0.18	-2.48	0.01

Table A55: Differences in trust between those assigned to the Ukrainian national identity affirmation treatment and those assigned to the control exercise (NIA, all assigned; C/IE = 4 consecutive)

	NIA=0	NIA=1	Difference	t-statistic	p-value
Trust Russian Gov (2 items, full sample)	-0.04	0.06	-0.09	-1.70	0.09
Trust Russian Gov (2 items, West-Center)	-0.16	-0.17	0.02	0.32	0.75
Trust Russian Gov (2 items, Southeast)	0.14	0.37	-0.23	-2.42	0.02
Trust Russian Ppl (2 items, full sample)	0.11	0.14	-0.03	-0.73	0.47
Trust Russian Ppl (2 items, West-Center)	0.00	-0.03	0.03	0.51	0.61
Trust Russian Ppl (2 items, Southeast)	0.27	0.38	-0.11	-1.42	0.16
Trust (4 items, full sample)	0.06	0.13	-0.07	-1.63	0.10
Trust (4 items, West-Center)	-0.05	-0.07	0.03	0.53	0.60
Trust (4 items, Southeast)	0.23	0.41	-0.19	-2.56	0.01

10.2.3 Regional differences in failure/refusal to engage with treatments

Table A56: Regional differences in failure/refusal to engage with treatments

	West-Center	Southeast	Difference	t-statistic	p-value
Failure/Refusal to engage w/OIA	0.46	0.40	0.06	1.19	0.24
Failure/Refusal to engage w/NIA	0.40	0.51	-0.11	-2.55	0.01
Failure/Refusal to engage w/Control	0.42	0.41	0.00	0.01	0.99

Note: Note: OIA = overarching Eastern Slavic identity affirmation treatment; NIA = national Ukrainian identity affirmation treatment. Failure/refusal to engage with treatments/control is coded as 1 if the respondent answered that the affirmation exercise made them think about values unimportant to Eastern Slavs/Ukrainians or if the respondent explicitly said 'refuse to answer' or if the respondent kept quiet during the manipulation check question. Failure/refusal to engage with treatments/control is coded as 0 if the respondent passed the manipulation check. Careless or insufficient effort (C/IE) responses are defined as three identical consecutive values in the outcome measures

11 How the KIIS determines convenient language of communication

The KIIS randomizes the language in which each respondent is greeted. While they have not saved the records of which respondent was greeted in which language, they report that approximately half of respondents were greeted in Ukrainian and half—in Russian. We have, however, the record of in which language respondents answered, i.e., in which language the rest of the interview was conducted:

- 51.55% clearly stated that they preferred Ukrainian,
- 38.65% stated that they preferred Russian,
- 4.65% said that there is no difference for them but they speak Ukrainian more often,
- 0.9% answered in Ukrainian "it's hard to say which language is more convenient" (and the interview was conducted in Ukrainian),
- 2.65% aid that there is no difference for them but they speak Russian more often,
- 1.6% answered in Russian "it's hard to say which language is more convenient" (and the interview was conducted in Russian).

In total, 57.1% of respondents continued the interview in Ukrainian and 42.9%—in Russian.

12 Randomization: Are primary variables and demographics balanced between included and excluded observations?

Table A57: Differences between quality and careless and insufficient effort (C/IE) observations

	C/IE=0	C/IE=1	Difference	t-statistic	p-value
Trust twd Gov	0.11	-0.61	0.72	11.39	0.00
Trust twd Ppl	0.18	-1.31	1.50	28.80	0.00
Overall Trust	0.14	-0.95	1.10	22.22	0.00
Age	47.10	48.70	-1.60	-1.39	0.16
Female	1.55	1.44	0.11	3.24	0.00
Education	6.59	6.52	0.07	0.63	0.53
Income	2.60	2.74	-0.14	-2.22	0.03
Southeast	0.40	0.27	0.14	4.03	0.00
Thermometer twd Russia	-0.51	-26.35	25.84	9.99	0.00
Would vote none	0.38	0.33	0.05	1.55	0.12
Professional	0.23	0.23	-0.01	-0.28	0.78
Retired	0.25	0.27	-0.02	-0.54	0.59
Settlement Type	4.80	4.90	-0.10	-0.66	0.51
Russian language	0.45	0.28	0.17	4.79	0.00
Russian ethnicity	0.06	0.01	0.04	2.76	0.01

Note: Careless or insufficient effort (C/IE) responses are defined as three identical consecutive values in the outcome measures

Table A58: Differences between quality and careless and insufficient effort (C/IE) observations

	C/IE=0	C/IE=1	Difference	t-statistic	p-value
Trust twd Gov	0.08	-0.63	0.72	9.56	0.00
Trust twd Ppl	0.14	-1.44	1.58	24.99	0.00
Overall Trust	0.11	-1.04	1.15	19.40	0.00
Age	47.10	49.37	-2.27	-1.69	0.09
Female	1.55	1.41	0.14	3.35	0.00
Education	6.57	6.67	-0.10	-0.76	0.45
Income	2.60	2.77	-0.17	-2.25	0.02
Southeast	0.40	0.27	0.13	3.14	0.00
Thermometer twd Russia	-1.31	-28.24	26.93	8.91	0.00
Would vote none	0.38	0.31	0.06	1.58	0.11
Professional	0.23	0.25	-0.02	-0.61	0.54
Retired	0.25	0.29	-0.04	-1.07	0.28
Settlement Type	4.80	4.99	-0.20	-1.07	0.28
Russian language	0.44	0.28	0.16	4.07	0.00
Russian ethnicity	0.05	0.01	0.04	2.32	0.02

Note: Careless or insufficient effort (C/IE) responses are defined as four identical consecutive values in the outcome measures

Table A59: Differences between respondents who passed/engaged with the MC after the OIA treatment and those who failed the OIA treatment or refused to engage with the MC after the OIA treatment

	Refused OIA treat=0	Refused OIA treat=1	Difference	t-statistic	p-value
Trust twd Gov	0.04	0.05	-0.01	-0.07	0.94
Trust twd Ppl	0.01	0.02	-0.01	-0.17	0.86
Overall Trust	0.03	0.05	-0.02	-0.33	0.74
Age	46.91	47.99	-1.08	-0.81	0.42
Female	1.52	1.51	0.00	0.10	0.92
Education	6.41	6.89	-0.47	-3.71	0.00
Income	2.55	2.74	-0.19	-2.68	0.01
Southeast	0.37	0.33	0.05	1.16	0.25
Thermometer twd Russia	-5.28	-3.25	-2.04	-0.64	0.52
Would vote none	0.37	0.36	0.02	0.39	0.70
Professional	0.21	0.28	-0.07	-2.01	0.05
Retired	0.27	0.23	0.04	1.23	0.22
Settlement Type	4.65	5.21	-0.56	-3.13	0.00
Russian language	0.44	0.39	0.05	1.17	0.24
Russian ethnicity	0.04	0.05	-0.00	-0.24	0.81

Table A60: Differences between respondents who passed/engaged with the MC after the NIA treatment and those who failed the NIA treatment or refused to engage with the MC after the NIA treatment

	Refused NIA treat=0	Refused NIA treat=1	Difference	t-statistic	p-value
Trust twd Gov	0.01	0.10	-0.09	-0.99	0.32
Trust twd Ppl	0.02	-0.03	0.05	0.63	0.53
Overall Trust	0.02	0.03	-0.00	-0.07	0.95
Age	46.37	48.14	-1.77	-1.26	0.21
Female	1.55	1.58	-0.03	-0.74	0.46
Education	6.62	6.73	-0.10	-0.75	0.46
Income	2.60	2.63	-0.03	-0.38	0.70
Southeast	0.36	0.49	-0.13	-3.02	0.00
Thermometer twd Russia	-0.30	-3.74	3.44	1.02	0.31
Would vote none	0.37	0.45	-0.08	-1.90	0.06
Professional	0.26	0.21	0.05	1.45	0.15
Retired	0.22	0.27	-0.05	-1.41	0.16
Settlement Type	4.67	5.09	-0.42	-2.19	0.03
Russian language	0.41	0.48	-0.07	-1.54	0.12
Russian ethnicity	0.05	0.06	-0.01	-0.74	0.46

Table A61: Differences between respondents who passed/engaged with the MC after control and those who failed the MC after control or refused to engage with the MC after control

	Refused Control=0	Refused Control=1	Difference	t-statistic	p-value
Trust twd Gov	0.03	-0.11	0.14	2.02	0.04
Trust twd Ppl	0.05	-0.11	0.15	2.19	0.03
Overall Trust	0.04	-0.12	0.16	2.72	0.01
Age	44.69	50.26	-5.57	-4.31	0.00
Female	1.56	1.49	0.07	1.64	0.10
Education	6.64	6.39	0.25	1.94	0.05
Income	2.69	2.51	0.17	2.41	0.02
Southeast	0.40	0.38	0.02	0.52	0.60
Thermometer twd Russia	-4.90	-3.02	-1.88	-0.61	0.54
Would vote none	0.34	0.38	-0.05	-1.22	0.22
Professional	0.22	0.21	0.01	0.38	0.70
Retired	0.21	0.32	-0.12	-3.36	0.00
Settlement Type	4.71	4.87	-0.16	-0.89	0.37
Russian language	0.45	0.39	0.05	1.34	0.18
Russian ethnicity	0.05	0.04	0.01	0.42	0.67
